# Linear ubiquitin chain assembly complex coordinates late thymic T-cell differentiation and regulatory T-cell homeostasis

**DOI:** 10.1038/ncomms13353

**Published:** 2016-11-18

**Authors:** Charis E. Teh, Najoua Lalaoui, Reema Jain, Antonia N. Policheni, Melanie Heinlein, Silvia Alvarez-Diaz, Julie M. Sheridan, Eva Rieser, Stefanie Deuser, Maurice Darding, Hui-Fern Koay, Yifang Hu, Fiona Kupresanin, Lorraine A. O'Reilly, Dale I. Godfrey, Gordon K. Smyth, Philippe Bouillet, Andreas Strasser, Henning Walczak, John Silke, Daniel H. D. Gray

**Affiliations:** 1 The Walter and Eliza Hall Institute of Medical Research, 1G Royal Parade, Parkville, Victoria 3052, Australia; 2 Department of Medical Biology, The University of Melbourne, Melbourne, Victoria 3052, Australia; 3 Centre for Cell Death, Cancer and Inflammation, University College London, London WC1E 6BT, UK; 4 The Department of Microbiology and Immunology, The Peter Doherty Institute for Infection and Immunity, The University of Melbourne, 792 Elizabeth Street, Melbourne, Victoria 3000, Australia; 5 The Australian Research Council Centre of Excellence for Advanced Molecular Imaging, The University of Melbourne, Melbourne, Victoria 3052, Australia; 6 Department of Mathematics and Statistics, The University of Melbourne, Melbourne, Victoria 3052, Australia

## Abstract

The linear ubiquitin chain assembly complex (LUBAC) is essential for innate immunity in mice and humans, yet its role in adaptive immunity is unclear. Here we show that the LUBAC components HOIP, HOIL-1 and SHARPIN have essential roles in late thymocyte differentiation, FOXP3^+^ regulatory T (Treg)-cell development and Treg cell homeostasis. LUBAC activity is not required to prevent TNF-induced apoptosis or necroptosis but is necessary for the transcriptional programme of the penultimate stage of thymocyte differentiation. Treg cell-specific ablation of HOIP causes severe Treg cell deficiency and lethal immune pathology, revealing an ongoing requirement of LUBAC activity for Treg cell homeostasis. These data reveal stage-specific requirements for LUBAC in coordinating the signals required for T-cell differentiation.

## Abstract (web summary)

LUBAC is a ubiquitin ligase complex of HOIL-1, HOIP and SHARPIN important for signal transduction of a range of stimuli. Here the authors define the function of all three LUBAC components in T cell development and homeostasis.

## Graphical abstract

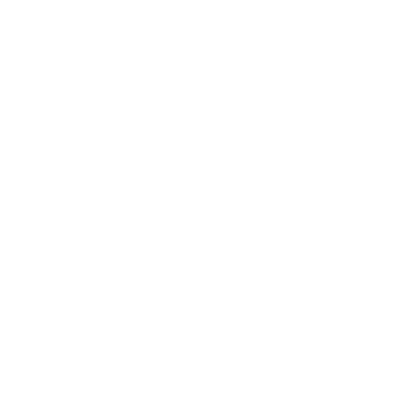

The thymus orchestrates the differentiation of haematopoietic precursors into diverse T-cell sub-lineages. These lineages include conventional T-cell receptor (TCR)αβ CD4^+^ and CD8^+^ T cells, Forkhead box-P3^+^ (FOXP3^+^) regulatory T (Treg) cells, natural killer T (NKT) cells, TCRγδ T cells and CD8αα T cells. A major determinant of cell fate is the specificity of the newly rearranged TCR for major histocompatibility complex (MHC) or MHC-like molecules presenting self-constituents, yet this stimulus alone is not sufficient to elaborate the many different T-cell types. T-cell differentiation is also influenced by cytokine receptors, members of the tumour necrosis factor receptor (TNFR) superfamily, chemokine receptors and adhesion molecules. Yet, precisely how these various cues are integrated to coordinate T-cell differentiation is unclear.

Positive selection rescues double-positive (DP) thymocytes from death-by-neglect and initiates the largest transcriptional re-programming in T-cell differentiation^1^. The upregulation of the C–C chemokine receptor type 7 (CCR7) mediates the migration of thymocytes from the cortex to the medulla as they differentiate into CD4^+^ or CD8^+^ single-positive (SP) cells. During residency in the medulla^2^, SP thymocytes undergo further maturation that involves a switch in TCR responses from apoptosis to proliferation and acquisition of the capacity to emigrate from the thymus^3^. Few of the stimuli that drive this maturation are known, although the nuclear factor-κB (NF-κB) pathway and interleukin (IL)-7 receptor signalling are important^3^,^4^,^5^.

Treg cells are a potent immune modulatory subset of CD4^+^ T cells that emerge during the late stage of thymocyte differentiation^6^. The integration of cues from the TCR, members of the TNFR superfamily and cytokine receptors (mainly the IL-2 receptor) culminate in the expression of the key transcription factor, FOXP3 (refs 7, 8). The NF-κB signalling pathway is critical for Treg cell differentiation, in particular, c-REL is necessary to consolidate FOXP3 expression to enable Treg cell proliferation^6^,^7^. In the periphery, Treg cells continue to rely on TCR and co-stimulatory inputs for their proliferation and differentiation into the various effector states that are required for proper immune regulation^9^,^10^,^11^.

The linear ubiquitin chain assembly complex (LUBAC) is composed of at least three proteins: ring finger protein 31 (RNF31/HOIP), RanBP-type and C3HC4-type zinc finger containing 1 (RBCK1/HOIL-1) and SHANK-associated RH domain interacting protein (SHARPIN/SIPL1)^12^. LUBAC can regulate diverse cell signalling pathways by catalysing the addition of linear ubiquitin chains to substrates. Innate and adaptive immune responses depend on LUBAC activity downstream of TNFR1, NOD2, TLR, NLRP3, TCR and B-cell receptor ligation^13^,^14^. These signals involve the linear ubiquitination of NEMO to reinforce canonical NF-κB signalling, although it is likely to be that other LUBAC substrates exist. Loss of LUBAC activity drives cells into apoptosis or necroptosis following exposure to TNF, lymphotoxin α or genotoxic stress^15^,^16^,^17^,^18^,^19^. All three LUBAC components are required for maximal linear ubiquitination; however, not all components are equal. Although HOIP deficiency alone completely ablates LUBAC activity^18^,^19^, SHARPIN-deficient cells still display substantial linear ubiquitination, because HOIL/HOIP complexes are able to sustain significant LUBAC function^17^,^18^,^19^. Consistent with these observations, HOIP-deficient mice are embryonic lethal^18^, whereas the SHARPIN-deficient mice from the chronic proliferative dermatitis mutation (*cpdm*) strain (hereafter referred to as *Sharpin*^*cpdm*^ mice) are born viable, but succumb to severe dermatitis at 12–14 weeks of age^20^,^21^.

Patients with loss-of-function mutations in *RBCK1* (encoding HOIL-1) or *RNF31* (encoding HOIP) exhibit impaired NF-κB responses, defects in B-cell activation and hyper-responsiveness of monocytes to IL-1β, the latter presumably driving auto-inflammatory disease^22^,^23^. These patients also had evidence of T-cell defects, including low thymic output and decreased TCRαβ^+^ CD4^+^ and CD8^+^ T cells, which exhibit poor proliferative responses to mitogens and antigens^22^,^23^, but whether these defects represent T-cell intrinsic defects is unclear.

In this study, we examine the requirement for each LUBAC component in T-cell and Treg cell lineages. The data reveal that LUBAC components play pivotal roles in late thymocyte differentiation of conventional T cells, non-conventional T cells and Treg cell homeostasis. LUBAC activity is necessary for the transcriptional programming of late thymocyte differentiation. Consistent with the distinct requirements for HOIL and HOIP versus SHARPIN in linear ubiquitination, the T-cell defects observed are more severe with HOIL-1 or HOIP deficiency compared with Sharpin deficiency. These data highlight previously unappreciated roles for LUBAC in T-cell biology.

## Results

### LUBAC activity is required for thymic T-cell differentiation

To determine whether T-cell differentiation requires LUBAC activity, we used loss-of-function genetic models for each of the three known components. We used a *Cd4*^*Cre*^ transgene to induce conditional excision of loxP-flanked alleles of *Rnf31* (the gene encoding HOIP) or *Rbck1* (the gene encoding HOIL-1) to create mice with T-lineage-specific deletion (hereafter termed *Hoip*^ΔCd4^ or *Hoil*^ΔCd4^, respectively). The role of the third LUBAC component was investigated using *Sharpin*^cpdm^ mice, which lack SHARPIN in all cells and were analysed before the development of extensive skin pathology.

**Figure 1:**
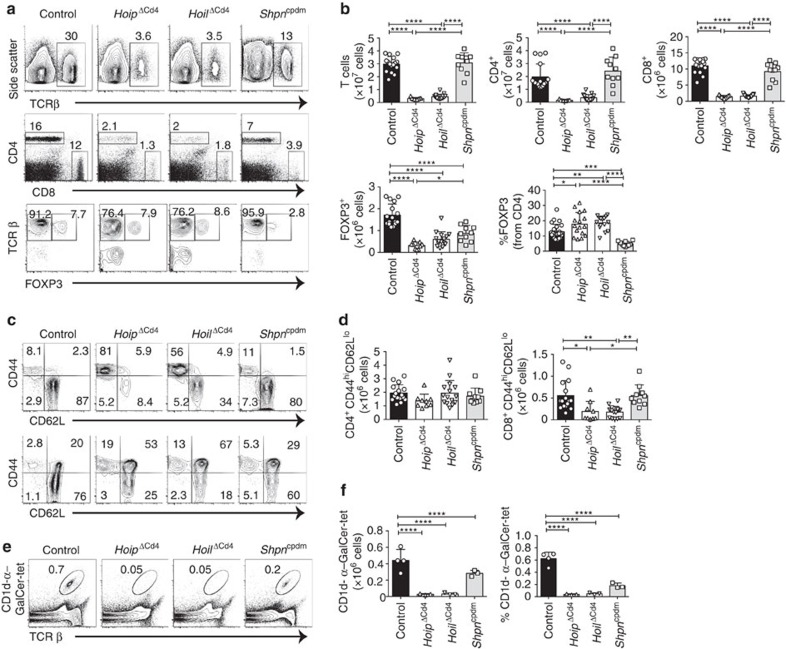
Absence of HOIP or HOIL-1 causes T-cell deficiency. (**a**) Flow cytometry of splenic cells from 7- to 15-week-old control, *Hoip*^ΔCd4^, *Hoil*^ΔCd4^ and *Sharpin*^cpdm^ assessed for expression of TCRβ (top panels), CD4 and CD8 (middle panels), and for FOXP3, after gating on CD4^+^ cells (bottom panels). (**b**) Quantification of CD4^+^CD8^+^TCRβ^+^ and CD4^+^FOXP3^+^ cells in the spleen. (**c**) Surface expression of CD44 and CD62L on CD4^+^FOXP3^−^ (top panels) and CD8^+^ T cells (bottom panels) in spleens of control, *Hoip*^ΔCd4^, *Hoil*^ΔCd4^ and *Sharpin*^cpdm^ mice. (**d**) Absolute numbers of CD44^high^CD62L^low^ activated cells in the CD4^+^FOXP3^−^ (left graph) and CD8^+^ (right graph) populations from the spleens of controls, *Hoip*^ΔCd4^, *Hoil*^ΔCd4^ and *Sharpin*^cpdm^ mice. (**e**) Flow cytometry of splenic CD1d-αGalCer tetramer-positive NKT cells from 7- to 15-week-old control, *Hoip*^ΔCd4^, *Hoil*^ΔCd4^ and *Sharpin*^cpdm^ mice. (**f**) Total cell numbers and percentages of CD1d-α-galactosylceramide (α-GalCer) tetramer-positive NKT cells in the spleen. For **b**,**d** and **f**, each symbol represents an individual mouse; small horizontal lines indicate mean±s.d.; *P<0.05, **P<0.01, ***P<0.005 and ****P<0.001, respectively. One-way analysis of variance with a Tukey's *post-hoc* test for multiple comparisons was used for statistical analysis. *Shpn*^cpdm^ refers to *Sharpin*^cpdm^ mice. Data are pooled from six independent experiments with two to six mice per group (**a**–**d**) or representative of two independent experiments with three to six mice per group (**e**,**f**).

The peripheral immune organs of *Hoip*^ΔCd4^ and *Hoil*^ΔCd4^ mice were almost completely devoid of CD8^+^ and CD4^+^ αβTCR^+^ T cells (Fig. 1a,b). Although the proportions of FOXP3^+^ Treg cells among αβTCR^+^ CD4^+^ T cells were normal in *Hoip*^ΔCd4^ and *Hoil*^ΔCd4^ mice, their number was greatly reduced, in line with the overall T-cell deficiency (Fig. 1a,b). The residual αβTCR^+^ T cells in *Hoip*^ΔCd4^ and *Hoil*^ΔCd4^ mice were predominantly CD44^hi^ CD62L^lo^ (Fig. 1c,d), suggestive of an activated/effector phenotype that is often associated with ‘homeostatic' expansion during lymphopenia^24^. By contrast, the numbers, proportions and activation status of conventional CD8^+^ and CD4^+^ T cells in *Sharpin*^cpdm^ mice were comparable to controls (Fig. 1a–d). Consistent with recent reports^25^,^26^, we observed that Treg cells were reduced in *Sharpin*^cpdm^ mice, although to a much lesser extent compared with the *Hoip*^ΔCd4^ and *Hoil*^ΔCd4^ mice (Fig. 1a,b). These data indicate that the LUBAC components HOIL-1 and HOIP are necessary for conventional CD4^+^ and CD8^+^ T-cell differentiation, and that SHARPIN is dispensable.

This differential requirement for LUBAC components extended to non-conventional T cells. CD1d-dependent NKT cells are potent immune modulatory T cells that can be detected by staining with α-galactosylceramide-loaded CD1d tetramers. NKT cells were virtually undetectable in the spleen and lymph nodes of *Hoip*^ΔCd4^ and *Hoil*^ΔCd4^ mice, yet they could be recovered from *Sharpin*^*cpdm*^ mice (albeit in reduced numbers compared to controls) (Fig. 1e,f and data not shown). Normal numbers of γδTCR^+^ T cells were found in *Sharpin*^cpdm^ mice (and, as expected, in *Hoip*^ΔCd4^ and *Hoil*^ΔCd4^ mice, as *Cd4*^*Cre*^ only becomes active after divergence of the αβTCR and γδTCR T cell lineages; Supplementary Fig. 1).

**Figure 2:**
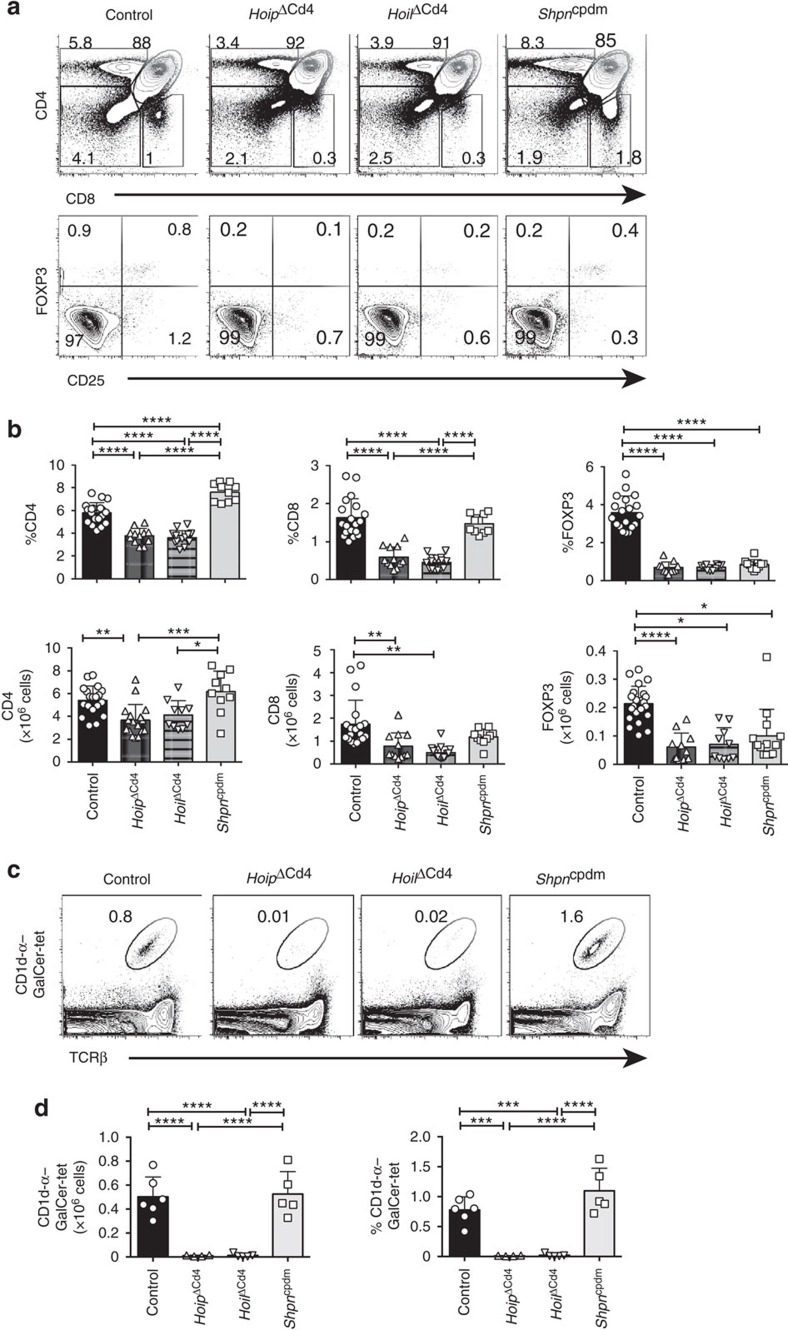
Thymic T-cell differentiation in LUBAC-deficient mice. (**a**) Surface staining of CD4 and CD8 (upper panels), and FOXP3 and CD25 (lower panels) on thymocytes from 7- to 15-week-old control, *Hoip*^ΔCd4^, *Hoil*^ΔCd4^ and *Sharpin*^cpdm^ mice. (**b**) Quantification of total cell numbers and percentages of CD4^+^ CD8^+^ and FOXP3^+^ thymocytes. (**c**) Flow cytometric analysis of thymic CD1d-αGalCer tetramer-positive NKT cells from 7- to 15-week-old control, *Hoip*^ΔCd4^, *Hoil*^ΔCd4^ and *Sharpin*^cpdm^ mice. (**d**) Total cell numbers and percentages of CD1d-αGalCer tetramer-positive NKT cells in the spleen. For **b** and **d**, each symbol represents an individual mouse; small horizontal lines indicate mean±s.d.; *P<0.05, **P<0.01, ***P<0.005 and ****P<0.001, respectively. One-way analysis of variance with a Tukey's *post-hoc* test for multiple comparisons was used for statistical analysis. *Shpn*^cpdm^ refers to *Sharpin*^cpdm^ mice. Data are pooled from six independent experiments with two to six mice per group (**a**,**b**) or representative of two independent experiments with four to six mice per group (**c**,**d**).

We tracked the origin of these T-cell defects to the thymus. The proportions of CD4^+^ and CD8^+^ SP thymocytes were significantly reduced in *Hoip*^ΔCd4^ and *Hoil*^ΔCd4^ mice but were found to be normal in *Sharpin*^cpdm^ mice (Fig. 2a,b and Supplementary Fig. 2). By contrast, the proportions and numbers of FOXP3^+^ Treg cells among CD4SP thymocytes were greatly diminished in all three strains, as were the CD25^+^ FOXP3^−^ and CD25^−^ FOXP3^+^ thymic Treg cell precursors (Fig. 2a,b). These data demonstrate that all three LUBAC components are required for the earliest checkpoint in Treg cell differentiation. NKT cells were almost undetectable in the thymus of *Hoip*^ΔCd4^ and *Hoil*^ΔCd4^ mice but were present in normal numbers in *Sharpin*^cpdm^ mice (Fig. 2c,d), demonstrating that this lineage has a dependency on LUBAC similar to that of conventional αβTCR T cells.

### Late-stage thymocyte differentiation requires HOIL-1 and HOIP

**Figure 3:**
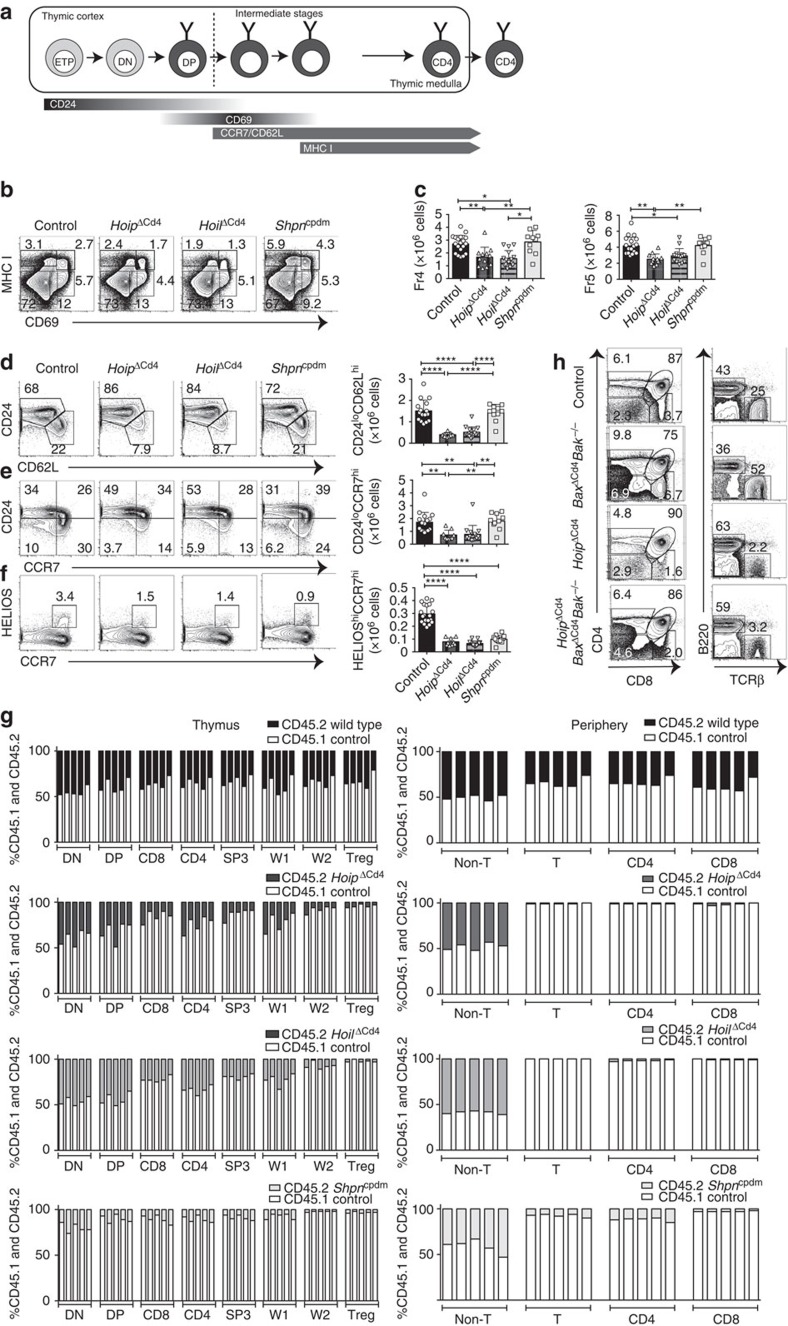
SP thymocyte differentiation and survival in LUBAC-deficient mice. (**a**) Schematic representation of CD4+ T-cell development in the thymus. Earliest thymic progenitor (ETP) cells undergo progressive differentiation from DN to DP to single-positive (CD4 or CD8) cell. The different stages of thymocyte development are also accompanied by changes in CD24, CD62L, CCR7, CD69 and MHC I (H2-K^b^) surface marker expression on the differentiating thymocyte. (**b**) Flow cytometric analysis of the surface expression of MHC I (H2-K^b^) versus CD69 on whole thymocytes from 7- to 15-week-old control, *Hoip*^ΔCd4^, *Hoil*^ΔCd4^ and *Sharpin*^cpdm^ mice. (**c**) Quantification of total cell numbers and percentages of Fraction 4 (MHC I^high^ CD69^high^) and Fraction 5 (MHC I^high^ CD69^low^) populations for control, *Hoip*^ΔCd4^, *Hoil*^ΔCd4^ and *Sharpin*^cpdm^ mice. Surface expression of CD24 versus CD62L (**d**), CD24 versus CCR7 (**e**), Helios versus CCR7 (**f**) gated on CD4SP from 7- to 15-week-old control, *Hoip*^ΔCd4^, *Hoil*^ΔCd4^ and *Sharpin*^cpdm^ mice. Right, cell numbers of the mature CD24^low^ CD62L^high^, CD24^low^ CCR7^high^ and HELIOS^high^ CCR7^high^ from CD4SP cells. (**g**) Contribution to different thymic T cell subsets in 50:50 mixed bone marrow chimeras 8 weeks after reconstitution. Columns show percentage of WT CD45.1^+^ (white bar) and CD45.2^+^ control, *Hoip*^ΔCd4^, *Hoil*^ΔCd4^ and *Sharpin*^cpdm^ (black bar) cells in individual chimeric mice. DN; CD4^−^CD8^−^, DP; CD4^+^CD8^+^ SP3; CD4^+^CD24^low^CCR7^high^, W1; CD4^+^CCR7^low^,HELIOS^+^, W2; CD4^+^CCR7^high^,HELIOS^+^, Treg; CD4^+^FOXP3^+^. Each bar represents an individual mouse. (**h**) Surface expression of CD4 and CD8 on whole thymocytes (upper panels) and B220 and TCRβ on splenocytes (lower panels) from control, *Bax*^ΔCd4^*Bak*^−/−^, *Hoip*^ΔCd4^ and *Hoip*^ΔCd4^*Bax*^ΔCd4^*Bak*^−/−^ mice. For **c**,**d**,**e** and **f**, each symbol represents an individual mouse; small horizontal lines indicate mean±s.d.; *P<0.05, **P<0.01, ***P<0.005 and ****P<0.001, respectively. One-way analysis of variance with a Tukey's *post-hoc* test for multiple comparisons was used for statistical analysis. *Shpn*^cpdm^ refers to *Sharpin*^cpdm^ mice. Data are pooled from six independent experiments with two to six mice per group (**b**–**f**) or representative of two independent experiments with four to six mice per group (**g**), or representative of two independent experiments with one to five mice per group (**h**).

To parse out thymocyte differentiation following positive selection, we employed a staging scheme validated by Mingueneau *et al*.^1^ (schematically represented in Fig. 3a), comparing expression of the early activation marker CD69 and the mature SP marker MHC class I (MHC I, H2-K^b^). The proportions of pre-selection (CD69^−^ MHC I^low^) and early selection thymocytes (CD69^low/high^ MHC I^low^) were comparable among all strains (Fig. 3b), with normal numbers of CD4^+^CD8^low/intermediate^ cells^27^ progressing through differentiation (Supplementary Fig. 3). However, both *Hoip*^ΔCd4^ and *Hoil*^ΔCd4^ mice exhibited specific loss of the late selection/mature subsets (CD69^high^ MHC I^high^ and CD69^low^ MHC I^high^, termed Fractions 4 and 5, ref. 1), Fig. 3b and Supplementary Fig. 3). This defect corresponded to the loss of ‘mature' CD24^low^ CD62L^high^ CCR7^+/−^ CD4SP (Fig. 3c,d and Supplementary Fig. 3). By contrast, thymocytes from *Sharpin*^*cpdm*^ mice exhibited largely normal progression through these differentiation stages (Fig. 3b–e). These data establish that, in the absence of HOIL-1 or HOIP, early positive and negative selection events occur normally, but that SP thymocyte maturation is almost completely blocked.

Positively selected thymocytes upregulate the chemokine receptor CCR7 and migrate into the medulla, where a second ‘wave' of thymocyte selection and differentiation of FOXP3^+^ Treg cells occurs^7^,^28^. High expression of the transcription factor HELIOS in FOXP3^−^ CCR7^+^ CD4SP identifies thymocytes destined for either deletion (negative selection) or early differentiation into the Treg cell lineage following high-avidity TCR stimulation^29^. We noted a marked reduction in the proportions and numbers of HELIOS^high^ FOXP3^−^ CCR7^+^ CD4SP in *Hoip*^ΔCd4^, *Hoil*^ΔCd4^ and *Sharpin*^cpdm^ mice compared with controls (Fig. 3f). However, CD5 levels (a surrogate marker of TCR signal strength) in mice lacking LUBAC components were comparable to those seen in controls (data not shown). These data suggest that the reduction in HELIOS^high^ FOXP3^−^ CCR7^+^ CD4SP cells in mice lacking LUBAC components was not associated with altered TCR signal strength but rather impaired induction of differentiation programmes parallel to or downstream of high-avidity TCR signals.

Interactions between maturing thymocytes and thymic epithelial cells are essential for the induction of a normal thymic medulla, primarily via the provision of ligands for members of the TNFR superfamily that are expressed by the epithelium^30^. To investigate the possibility that defects in the thymic microenvironment might contribute to the block in SP thymocyte differentiation observed in *Hoip*^ΔCd4^ and *Hoil*^ΔCd4^ mice, we created irradiation chimeras reconstituted with 50:50 mixtures of haematopoietic precursors from the mutant mice with CD45.1^+^ congenically marked wild-type (WT) mice. Although the double-negative (DN) and DP thymocyte precursor populations showed ∼40–50% representation of the CD45.2^+^ *Hoip*^ΔCd4^ or *Hoil*^ΔCd4^ compartments, there was a specific loss of CD45.2^+^ cells at the CD8SP, CD4SP CCR7^+^ ‘wave 2' and FOXP3^+^ Treg cell stages (Fig. 3g). Virtually, no HOIP- or HOIL-1-deficient T-lineage cells were detected in the periphery of these chimeras (Fig. 3g). These data demonstrate that the requirement for LUBAC activity is T-cell intrinsic.

We then tested whether LUBAC deficiency was causing apoptosis of SP thymocytes. LUBAC activity might be required to prevent induction of pro-apoptotic BH3-only proteins or to upregulate pro-survival BCL-2 proteins downstream of TCR signalling^31^, or in response to DNA damage^15^. Therefore, we tested whether the complete ablation of the mitochondrial pathway of apoptosis would rescue T-cell differentiation in *Hoip*^ΔCd4^ mice. The multi-BH domain pro-apoptotic BCL-2 family proteins BAX and BAK are essential for the mitochondrial outer membrane permeabilization that executes this pathway of apoptosis^32^. Extensive redundancy between BAX and BAK, and the early lethality observed in *Bax*^−/−^*Bak*^−/−^ mice necessitated conditional deletion of *Bax* using *Cd4*^*Cre*^ on a *Bak*^−/−^ background to induce T-cell-specific ablation of the mitochondrial pathway of apoptosis. As expected, the DN block observed in *Bax*^−*/*−^*Bak*^−*/*−^ haematopoietic chimeras^33^ was bypassed in *Bax*^ΔCd4^*Bak*^−*/*−^ mice, yet expansion of CD4SP, CD8SP and mature DN thymocytes, and increased percentages of peripheral T cells were observed (Fig. 3h). The compound loss of BAX and BAK in *Hoip*^ΔCd4^ mice did not restore late-stage thymocyte differentiation or peripheral T cells (Fig. 3h and data not shown). These data indicate that LUBAC was neither required to antagonize thymocyte deletion nor to transduce pro-survival signals to block the mitochondrial apoptotic pathway.

### NF-κB signalling is partially impaired by LUBAC deficiency

**Figure 4:**
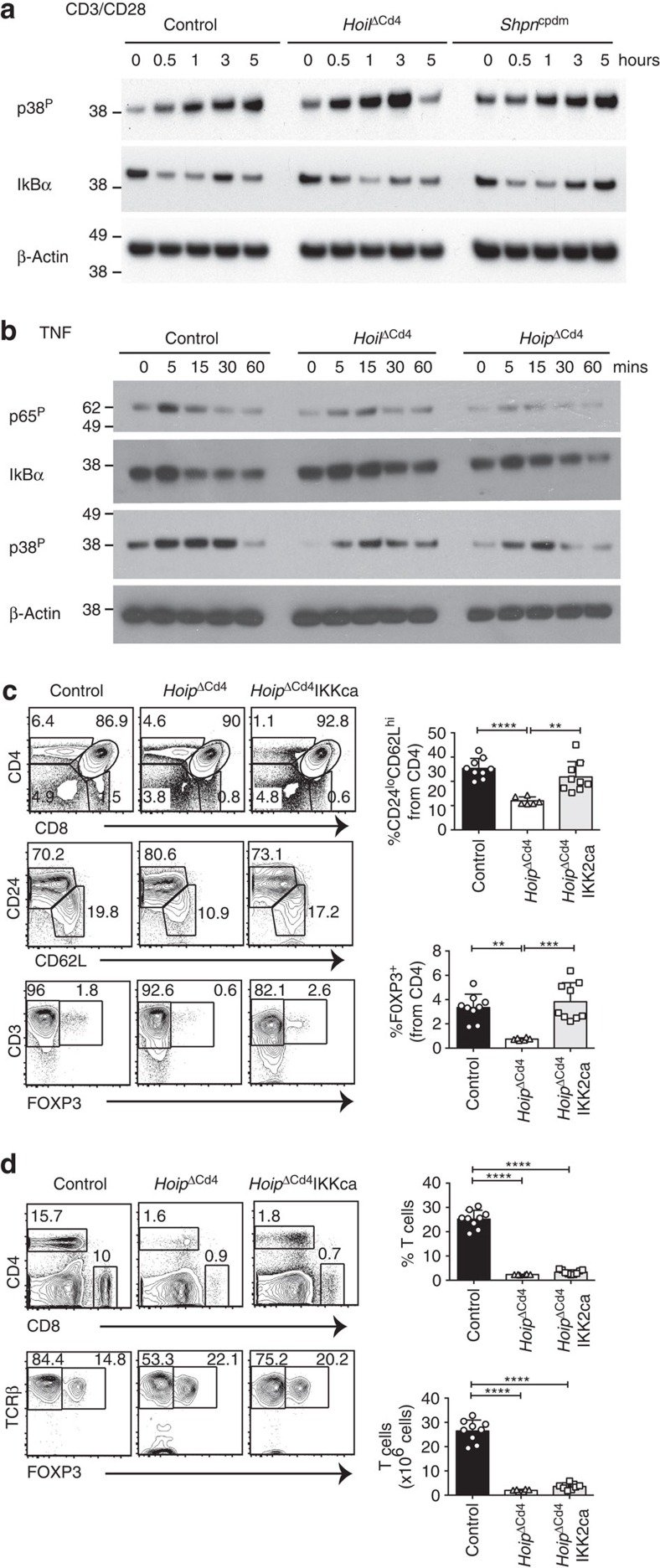
Perturbed TCR or TNFR signalling in LUBAC-deficient thymocytes. Immunoblot analysis of phosphorylated (activated) p38, phosphorylated (activated) p65 or total IκBα from thymocytes left unstimulated (0) or stimulated with anti-CD3/CD28 for 0.5, 1, 3 or 5 h (**a**) or with TNF for 5, 15, 30 or 60 min (**b**). (**c**) Surface staining of CD4 and CD8 (upper panels), CD24 and CD62L gated on CD4^+^ cells (middle panels), CD3 and FOXP3 gated on CD4^+^ cells (lower panels) and numerical quantification of thymocytes from 7- to 15-week-old control, *Hoip*^ΔCd4^ and *Hoip*^ΔCd4^IKKca mice. (**d**) Surface expression of CD4 and CD8 (upper panels), TCRβ and FOXP3 (lower panels) on splenocytes from control, *Hoip*^ΔCd4^ and *Hoip*^ΔCd4^IKKca mice. For **a** and **b**, data are representative of two independent experiments with one mouse per group. For **c** and **d**, each symbol represents an individual mouse; small horizontal lines indicate mean±s.d.; *P<0.05, **P<0.01, ***P<0.005 and ****P<0.001, respectively. One-way analysis of variance with a Tukey's *post-hoc* test for multiple comparisons was used for statistical analysis. Data are pooled from three independent experiments with two to five mice per group (**c**,**d**).

Previous studies found that LUBAC is critical for optimal NF-κB activation downstream of immune receptor signalling in B-cell lymphomas and Jurkat T cells by associating with the CARD11/BCL-10/MALT1 (CBM) complex^34^,^35^. Therefore, we compared the kinetics and extent of activation of the NF-κB pathway following CD3/CD28 stimulation of thymocytes from *Hoil*^ΔCd4^, *Sharpin*^cpdm^ and control mice. The degradation of inhibitor of κBα (IκBα) is a hallmark of NF-κB activation and was apparent within 0.5 h of CD3/CD28 stimulation of control thymocytes (Fig. 4a). However, in CD3/CD28-stimulated thymocytes from *Hoil*^ΔCd4^ mice, IκBα degradation was delayed (Fig. 4a), suggesting that loss of this LUBAC component caused defects in NF-κB activation. By contrast, the kinetics of p38 mitogen-activated protein kinase phosphorylation following CD3/CD28 stimulation of *Hoil*^ΔCd4^ and *Sharpin*^cpdm^ thymocytes was comparable to controls (Fig. 4a). Although these data show that LUBAC is involved in transducing TCR-dependent NF-κB signals in thymocytes, this defect is unlikely to explain the block in late-stage thymocyte differentiation observed in *Hoil*^ΔCd4^ and *Hoip*^ΔCd4^ mice. Loss of CARD11, BCL10 or MALT1 completely blocks NF-κB activation following TCR stimulation of thymocytes, yet these defects do not impair conventional T-cell development (for example, see ref. 36). Collectively, these findings suggest a requirement for LUBAC in thymocyte differentiation beyond NF-κB activation downstream of TCR and the CBM complex. Therefore, we tested whether LUBAC was also required in thymocytes for NF-κB activation downstream of stimulation of TNFR family members. TNF stimulation of WT thymocytes induced phosphorylation of p65 (RELA) within 5 min and degradation of IκBα within 15 min (Fig. 4b). HOIL-1 or HOIP deficiency reduced and delayed TNF-induced p65 phosphorylation and IκBα degradation (Fig. 4b). Although TNF-stimulated thymocytes from *Hoil*^ΔCd4^ and *Hoip*^ΔCd4^ mice exhibited similar kinetics of p38 mitogen-activated protein kinase phosphorylation, the overall levels appeared to be lower than in control cells (Fig. 4b). Collectively, these data reveal a requirement for LUBAC activity in optimal NF-κB activation downstream of both TCR and TNFR ligation.

To determine whether enforced NF-κB activation could rescue the thymic defects observed in *Hoip*^ΔCd4^ mice, we introduced a *Cre*-inducible allele of mutant *Ikbkb* that encodes a constitutively active form of IKK2 (IKKca) when expressed^37^. Although the proportions of CD4SP and CD8SP remained low in *Hoip*^ΔCd4^IKKca mice, the proportions of mature CD4^+^CD24^low^CD62^high^ cells and FOXP3^+^ cells were restored to levels observed in control mice (Fig. 4c). Nevertheless, *Hoip*^ΔCd4^IKKca mice had severe T-cell deficiency in the periphery (Fig. 4d). This outcome indicates that the reinforcement of NF-κB signalling in LUBAC-deficient T cells only partially rescues the block in late-stage T-cell differentiation, and that other cell survival or differentiation programmes must also rely on LUBAC.

### LUBAC does not antagonize TNF-induced killing of thymocytes

**Figure 5:**
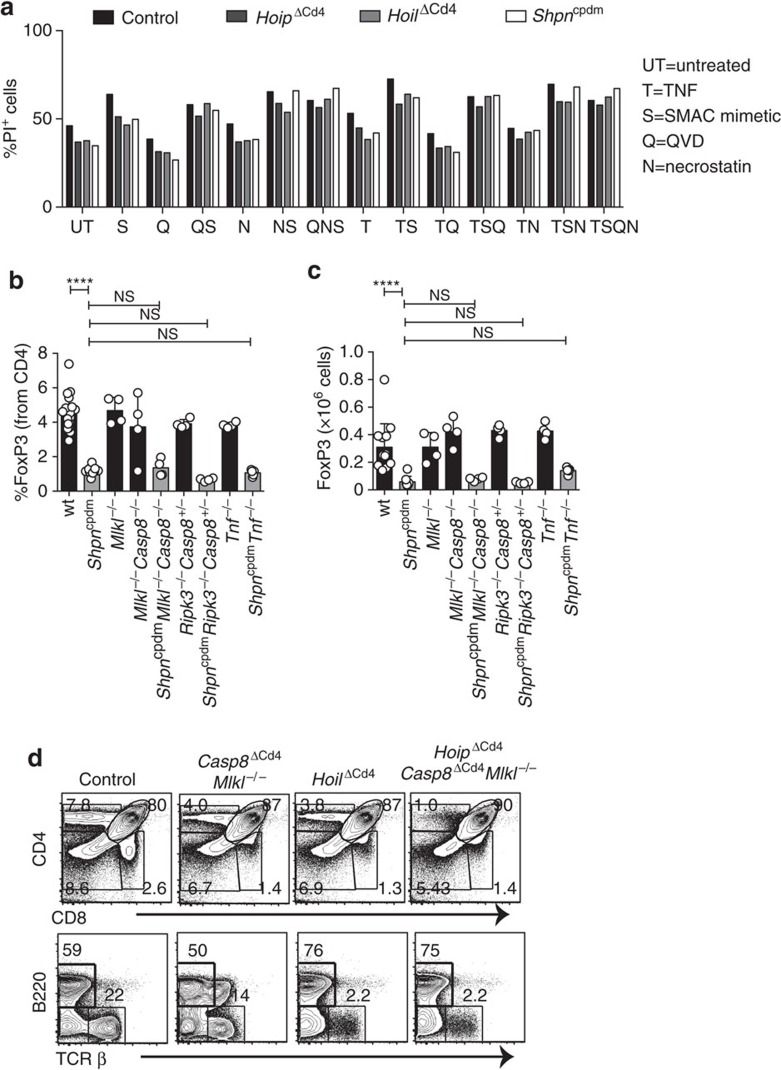
Inhibition of cell death in LUBAC-deficient thymocytes. (**a**) Cell death determined by PI uptake in thymocytes from control, *Hoip*^ΔCd4^, *Hoil*^ΔCd4^ and *Sharpin*^cpdm^ mice cultured for 24 h with combinations of agonists (T: 100 ng ml^−1^ TNF; S: 500 nM SMAC mimetic; Q:10 μM QVD-OPh;N: 10 μM necrostatin). Percentages (**b**) and absolute numbers (**c**) of CD4^+^FOXP3^+^ cells in the thymus of WT (*n*=18), *Sharpin*^cpdm^ (*n*=11), *Mlkl*^−/−^ (*n*=4), *Mlkl*^−/−^*Casp8*^−/−^ (*n*=4), *Sharpin*^cpdm^*Casp8*^−/−^*Mlkl*^−/−^ (*n*=4), *Rip3k*^−/−^*Casp8*^+/−^ (*n*=4), *Sharpin*^cpdm^*Rip3k*^−/−^*Casp8*^+/−^ (*n*=4), *Tnf*^−/−^ (*n*=4) and *Sharpin*^cpdm^*Tnf*^−/−^ (*n*=5) mice. *Shpn*^cpdm^ refers to *Sharpin*^cpdm^ mice. (**d**) Surface expression of CD4 and CD8 on whole thymocytes from control, *Casp8*^ΔCd4^*Mlkl*^−/−^, *Hoil*^ΔCd4^ and *Hoip*^ΔCd4^*Casp8*^Δcd4^*Mlkl*^−/−^mice. Data are representative of two independent experiments with one mouse per genotype (**a**,**d**). For **b** and **c**, each symbol represents an individual mouse, with 4–14 mice per group; small horizontal lines indicate mean±s.d.; *P<0.05, **P<0.01, ***P<0.005 and ****P<0.001, respectively. One-way analsyis of variance with a Tukey's *post-hoc* test for multiple comparisons was used for statistical analysis.

Impaired LUBAC function can switch pro-survival TNFR1 signalling into caspase-8-dependent apoptosis or caspase-independent, RIPK1/RIPK3/MLKL-mediated necroptosis^17^,^38^,^39^. TNF is produced constitutively in the thymic medulla by epithelial cells and dendritic cells^8^, prompting the hypothesis that LUBAC deficiency might lead to the death of medullary SP and Treg cells. Surprisingly, we found that TNF treatment of thymocytes from *Hoip*^ΔCd4^, *Hoil*^ΔCd4^ and *Sharpin*^cpdm^ mice did not induce greater cell death than observed in control thymocytes (Fig. 5a). Moreover, treatment of cells with TNF plus a small molecular mimetic of second mitochondria-derived activator of caspases (SMACs) did not induce additional death of *Hoip*^ΔCd4^, *Hoil*^ΔCd4^ and *Sharpin*^cpdm^ thymocytes. Similarly, when TNF/SMAC mimetic-induced cell death was blocked by the caspase inhibitor, QVD-OPh, to engage the alternative cell death mechanism, necroptosis, the viability of thymocytes from *Hoip*^ΔCd4^, *Hoil*^ΔCd4^ and *Sharpin*^cpdm^ mice was comparable to WT thymocytes (Fig. 5a). Necroptosis is dependent on the activities of RIPK1, RIPK3 and the pseudo-kinase MLKL. Blocking necroptosis using the RIPK1 inhibitor necrostatin-1 did not alter the survival of thymocytes from *Hoip*^ΔCd4^, *Hoil*^ΔCd4^ or *Sharpin*^cpdm^ mice *in vitro*. These data do not support the notion that LUBAC deficiency sensitizes thymocytes to TNF-induced cell death.

We also tested this hypothesis *in vivo* by analysing whether the loss of thymic Treg cells in *Sharpin*^cpdm^ mice could be rescued by genetic ablation of TNF or critical cell death inducers. *Tnf*^−/−^*Sharpin*^cpdm^ mice are protected from multi-organ inflammation^19^, but the fivefold reduction in thymic Treg cells caused by SHARPIN deficiency was not restored by loss of TNF (Fig. 5b,c). However, lymphotoxin α can serve as an alternative ligand for TNFR1 and is also extensively expressed in the thymic medulla. Formation of the death-inducing complex II following TNFR1 ligation can initiate caspase-8-dependent apoptosis or, when caspases are inhibited, RIPK3- and MLKL-dependent necroptosis^40^. Although normal differentiation of thymic Treg cells was observed in *Mlkl*^−/−^ and *Mlkl*^−/−^*Casp8*^−/−^ mice, the reduction of thymic Treg cells caused by SHARPIN deficiency was not corrected in *Sharpin*^cpdm^*Mlkl*^−/−^*Casp8*^−/−^ mice (Fig. 5b,c). Likewise, loss of *Ripk3* and *Casp8* haploinsufficiency failed to rescue the thymic Treg cell defects in *Sharpin*^cpdm^ mice (Fig. 5b,c).

To test whether the block in conventional thymocyte differentiation observed in *Hoip*^ΔCd4^ mice was caused by complex II-mediated apoptotic cell death, we generated *Hoip*^ΔCd4^*Casp8*^ΔCd4^*Mlkl*^−/−^ mice. Genetic ablation of caspase-8-mediated apoptosis and MLKL-mediated necroptosis failed to restore normal SP thymocyte differentiation or peripheral T-cell numbers in *Hoip*^ΔCd4^ mice (Fig. 5d). Collectively, these data demonstrate that LUBAC is not solely required to prevent apoptotic or necroptotic cell death in medullary thymocytes, but rather must be necessary for a process that is critical for the differentiation of maturing thymocytes.

### LUBAC is required for transcriptional programming of T cells

To identify the impact of LUBAC deficiency on the transcriptional programme of thymocyte differentiation immediately following positive selection, we fluorescence-activated cell sorting (FACS) purified lineage-depleted CD69^+^ MHC I^low^ and CD69^+^ MHC I^high^ thymocytes (Fractions 3 and 4; ref. 1) from WT, *Hoil*^ΔCd4^ and *Sharpin*^cpdm^ mice, and subjected them to RNA sequencing. These populations were selected because: (1) this transition is associated with consolidation of the large transcriptional changes that follow positive selection^1^; (2) the subset composition of these fractions, defined by CD4 and CD8 expression, was comparable among mice of the different genotypes (Supplementary Fig. 3); and (3) this transition immediately precedes the loss of mature SP observed in the *Hoil*^ΔCd4^ and *Hoip*^ΔCd4^ mice (Fig. 3a).

**Figure 6:**
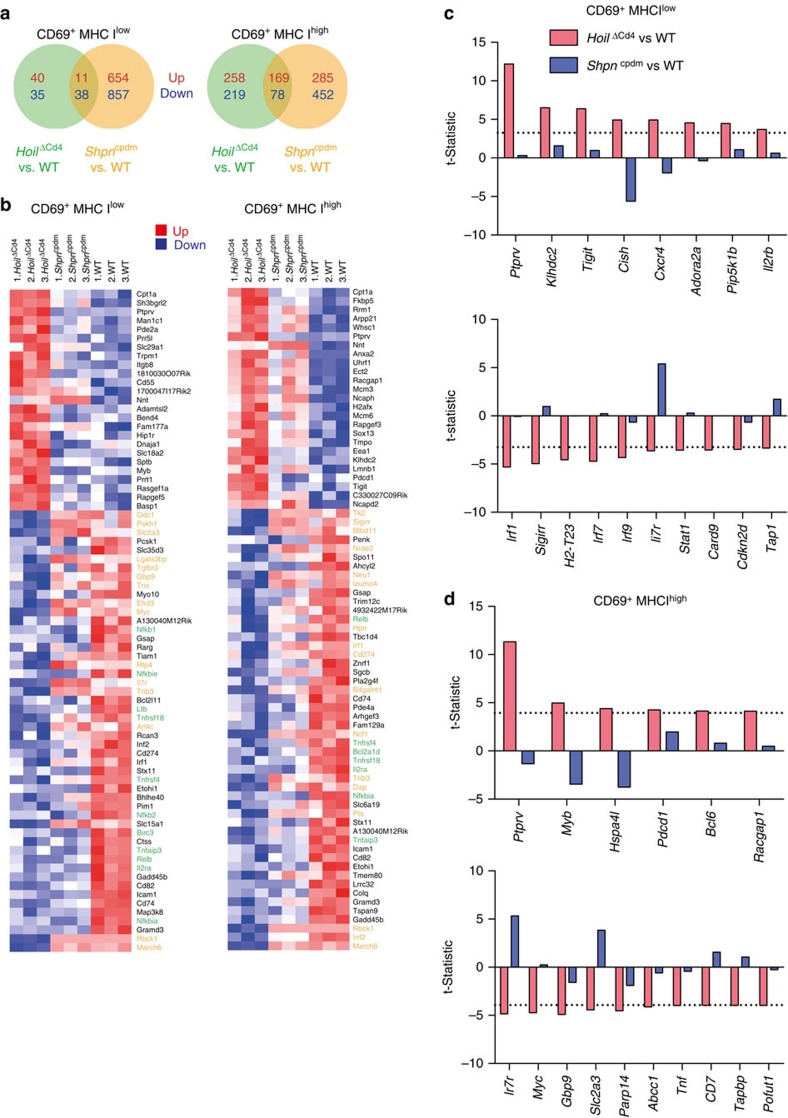
Transcriptional impact of LUBAC deficiency on T-cell differentiation. (**a**) Venn diagrams of the numbers of genes upregulated (red) or downregulated (blue) in comparisons of CD69^+^ MHC I^low^ or CD69^+^ MHC I^high^ thymocytes from *Hoil*^ΔCd4^ versus WT (green) and *Sharpin*^cpdm^ versus WT (orange) at a 5% false discovery rate (FDR) cutoff. (**b**) Heatmaps of individual log-expression values. Left plot shows the 25 most upregulated genes and 50 most downregulated genes for *Hoil*^ΔCd4^ versus WT in CD69^+^ MHC I^low^ thymocytes. Right plot show the same for CD69^+^ MHC I^high^. Genes are ordered by *P*-value. Red indicates relatively higher expression and blue indicates relatively lower expression. Genes highlighted in green are involved in NF-κB signalling, those in yellow are involved in thymocyte/Treg cell differentiation. *Shpn*^cpdm^ refers to *Sharpin*^cpdm^ mice. (**c**,**d**) Genes that are differentially expressed in *Hoil*^ΔCd4^ versus WT but show no change or opposite change in *Sharpin*^cpdm^. Results for CD69^+^ MHC I^low^ thymocytes are shown in **c** and CD69^+^ MHC I^high^ thymocytes in **d**. The plot shows the limma *t*-statistics for each gene for assessing differential expression; the dotted line indicates the 5% FDR cutoffs of *t*=3.25 for **c** and *t*=3.94 for **d**.

HOIL deficiency altered the transcriptome immediately following positive selection (124 differentially expressed genes in CD69^+^ MHC I^low^ thymocytes) and this effect was amplified in CD69^+^ MHC I^high^ thymocytes (724 differentially expressed genes; Fig. 6a). Surprisingly, thymocytes from *Sharpin*^cpdm^ mice had more substantial alteration of the transcriptome, perhaps reflecting the consequences of moderate LUBAC defects throughout T-cell differentiation (compared with the conditional deletion of HOIL-1 at the DP stage) and minor differences in the genetic background (still largely C57BL/Ka versus WT C57BL/6). To analyse the transcriptional changes associated with the block in SP thymocyte differentiation, we performed a heat-map analysis of the most significantly upregulated (25) or downregulated (50) genes in CD69^+^ MHC I^low^ and CD69^+^ MHC I^high^ thymocytes from *Hoil*^ΔCd4^ compared with WT mice (Fig. 6b). Reduced expression of many core NF-κB target genes was a common feature of thymocytes from *Hoil*^ΔCd4^ and *Sharpin*^cpdm^ mice. These included genes involved in negative feedback (for example, *Nfkbia* (or IκBα), *Nfkbie* (or IκBɛ), *Birc3* (or cIAP2) and *Tnfaip3* (or A20)) and T-cell differentiation (particularly of FOXP3^+^ Treg cells; for example, *Gadd45b*, *Il2ra*, *Tnfrsf18*, *Tnfrsf4* (or OX40) and *Relb*; Fig. 6b). To determine whether there was a defect in the induction of NF-κB target genes in the LUBAC-deficient strains during the differentiation from CD69^+^ MHC I^low^ into CD69^+^ MHC I^high^ thymocytes, we first identified 154 NF-κB target genes that were significantly up- or downregulated during this transition in WT cells. Barcode enrichment plots show that, despite the dampened transcription of NF-κB target genes, the magnitude and direction of changes induced in these transcripts during the post-positive selection stages was maintained in HOIL- and SHARPIN-deficient cells (Supplementary Fig. 4). These findings support our earlier data showing that, although LUBAC-deficient thymocytes have impaired NF-κB signalling, this defect does not by itself explain the block in thymocyte differentiation observed in *Hoil*^ΔCd4^ and *Hoip*^ΔCd4^ mice.

We therefore focused on transcriptional changes that were observed in thymocytes from *Hoil*^ΔCd4^ mice (where differentiation was blocked), but not *Sharpin*^cpdm^ mice (where conventional thymocyte differentiation proceeds normally). This filter revealed that genes involved in cytokine signalling were prominent; HOIL-deficient thymocytes failed to upregulate *Il7r* and downregulate *Cish* (encoding the SOCS family member, CIS, an inhibitor of IL-2 signalling), *Il2rb* and *Cxcr4* (Fig. 6c,d). Consistent with these data, HOIL-deficient thymocytes appeared to have a specific defect in an IL-2-sensitive glycolytic transcriptional programme, with heightened expression of Bcl6 and reduced Myc and Slc2a3 (a regulator of glycolysis), an apparent parallel with a recent study in mature T-cell differentiation^41^. The interferon signalling pathway was also selectively impaired in HOIL-deficient thymocytes, with reduced transcription of *Stat1*, *Irf1*, *Irf7* and *Irf9* in CD69^+^ MHC I^low^ cells (Fig. 6c). Previous studies demonstrating that type I interferon is a feature of late thymocyte differentiation^5^, and that *Irf1* is important for thymocyte differentiation^42^,^43^, suggest that these changes may also be critical for the impaired thymocyte development seen in the HOIL- and HOIP-deficient mice.

In summary, post-positive selection thymocytes from the LUBAC-deficient strains shared baseline defects in the transcription of NF-κB targets that may explain the observed Treg cell deficiency. Furthermore, the specific loss of transcripts in a number of essential signal transduction pathways from HOIL-1-deficient cells is likely to account for their block in late-stage thymic T-cell differentiation.

### LUBAC is essential for Treg cell homeostasis

**Figure 7:**
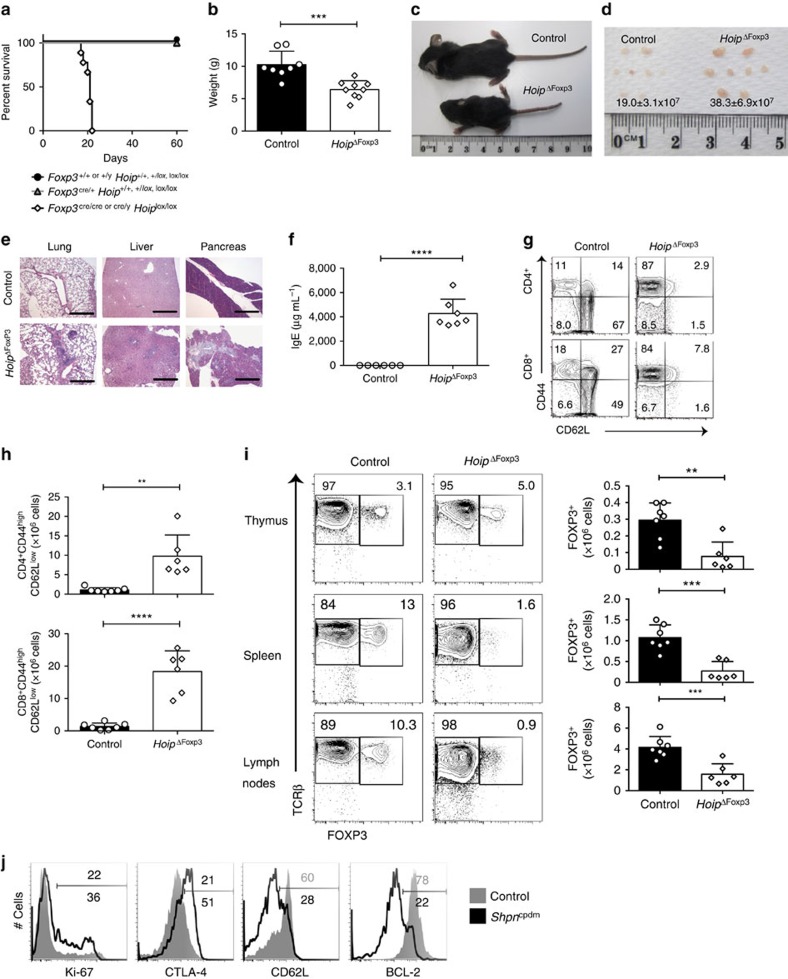
LUBAC is required for peripheral Treg cell homeostasis and tolerance. (**a**) Kaplan–Meyer survival curve measured from birth to 60 days for mice of the indicated genotypes (*P*<0.0001 using the log-ranked Mantel–Cox test). (**b**) Weights of 18–22-day-old male and female *Hoip*^ΔFoxp3^ mice and healthy littermate controls. (**c**) Runted appearance and (**d**) lymphadenopathy observed in *Hoip*^ΔFoxp3^ mice, with mean cell number indicated. (**e**) Representative hematoxylin and eosin (haematoxylin and eosin) stained sections of lung, liver and pancreas of 21-day-old mice of the indicated genotypes (scale bars, 500 μm). (**f**) Plasma IgE concentrations in 18–22-day-old *Hoip*^ΔFoxp3^ mice and healthy littermate controls. (**g**) Expression of CD44 and CD62L on CD4^+^FOXP3^−^ (top panel) and CD8^+^ T cells (bottom panel) in spleens of WT and *Hoip*^ΔFoxp3^ mice. (**h**) Absolute numbers of CD44^high^CD62L^low^ activated cells in the CD4^+^FOXP3^−^ and CD8^+^ population of WT and *Hoip*^ΔFoxp3^ mice. (**i**) Representative flow cytometry plots and absolute numbers of FOXP3^+^TCRβ^+^ cells in the thymus (top panel), spleen (middle panel) and lymph nodes (bottom panel) of WT and *Hoip*^ΔFoxp3^ mice. (**j**) Expression of Ki-67, CTLA-4, CD62L and BCL-2 in CD4^+^FOXP3^+^ cells in the spleen from WT (grey shaded histogram) and *Sharpin*^cpdm^ (black thick histogram) mice. *Shpn*^cpdm^ refers to *Sharpin*^cpdm^ mice. (*n*=6–9 per /genotype, bar graphs show mean±s.d.). *P<0.05, **P<0.01, ***P<0.005 and ****P<0.001, respectively. One-way analysis of variance with a Tukey's *post-hoc* test for multiple comparisons was used for statistical analysis. Data are pooled from (**a**,**b**,**f**,**g**,**h**) or representative (**c**–**e**) of three independent experiments with one to three mice per group.

The thymus of *Hoip*^ΔCd4^, *Hoil*^ΔCd4^ and *Sharpin*^cpdm^ mice all had equivalent reductions in ‘wave 2' cells, Treg cell precursors and mature Treg cells, yet only SHARPIN-deficient mice had substantial numbers of peripheral Treg cells (Figs 1 and 2). To determine whether the absence of Treg cells in *Hoip*^ΔCd4^ mice was merely secondary to the loss of conventional T cells in the periphery resulting in reduced levels of IL-2 (an essential cytokine for the maintenance of Treg cells) or whether this phenotype reflected an ongoing requirement for LUBAC activity for Treg cell homeostasis, we created male *Foxp3*^*Cre/y*^; *Rnf31*^fl/fl^ and female *Foxp3*^*Cre/Cre*^; *Rnf31*^fl/fl^ mice (both termed *Hoip*^ΔFoxp3^ hereafter). Mice of the control genotypes remained healthy and survived beyond 60 days of age, but all *Hoip*^ΔFoxp3^ mice developed a severe wasting disease and died around weaning (Fig. 7a–c). *Hoip*^ΔFoxp3^ mice exhibited severe immune pathology, including lymphadenopathy, lymphocytic perivascular infiltration and tissue destruction of the lung, liver and exocrine pancreas, hyper-IgE production and abnormally high numbers of activated CD4^+^ and CD8^+^ T cells (CD44^high^CD62L^low^KI-67^+^; Fig. 7d–h and Supplementary Fig. 5a–c). These features are all hallmarks of the *Foxp3*-deficient *scurfy* mouse phenotype^44^.

Although we observed normal proportions of thymic FOXP3^+^ Treg cells among CD4SP in *Hoip*^ΔFoxp3^ mice, there was a marked decrease in the numbers and proportions of CD4^+^ Treg cells in the spleen and lymph nodes (Fig. 7i and Supplementary Fig. 5d,e). To determine whether there was a cell intrinsic requirement for HOIP in peripheral Treg cells, we took advantage of the fact that the *Foxp3*^*YFP*−*Cre*^ knock-in allele is X-linked^45^ to create chimeras in heterozygous *Foxp3*^*Cre/+*^ females through X-inactivation. Although we could recover a substantial fraction of YFP^+^ Treg cells from the periphery of *Foxp3*^*Cre/+*^*Hoip*^*fl/+*^ control mice, very few YFP^+^ Treg cells were detected in *Foxp3*^*Cre/+*^*Hoip*^*fl/fl*^ females (Supplementary Fig. 5f,g). Treg cell loss in *Hoip*^ΔFoxp3^ mice could not be rescued by repeated anti-TNF treatment of neonatal mice commencing from day 5 (data not shown), suggesting that this defect was not caused by excessive TNF-induced cell death. These findings establish that ongoing LUBAC activity is required for the maintenance of mature Treg cells, with a greater reliance on HOIP activity than SHARPIN.

Nevertheless, a deeper examination of the peripheral Treg cell compartment of *Sharpin*^cpdm^ mice revealed substantial homeostatic perturbation, with markedly increased numbers of proliferating (Ki-67^+^) Treg cells, elevated expression of CTLA-4 (a key Treg cell effector molecule^46^), increased proportions of effector Treg cells (CD44^high^CD62L^low^) and reduced expression of the pro-survival protein, BCL-2 (Fig. 7j).

## Discussion

These data reveal an essential, cell intrinsic role for LUBAC in multiple aspects of T-cell differentiation. HOIP and HOIL-1 were required for the differentiation of conventional αβT cells, FOXP3^+^ Treg cells and NKT cells in the thymus (in *Hoip*^ΔCd4^ and *Hoil*^ΔCd4^ mice) and maintenance of Treg cells in the periphery (in *Hoip*^ΔFoxp3^ mice). These findings suggest that the T-cell deficiency observed in patients with loss-of-function mutations affecting HOIL and HOIP is a primary defect^22^,^23^. The thymic phenotypes caused by loss of HOIL or HOIP are reminiscent of those observed with T-cell-specific deletion of NEMO^47^ or TAK1 (refs 5, 48, 49) and align well with the interactions described between LUBAC and these components of NF-κB signalling^13^,^14^,^50^. However, constitutive activation of the NF-κB pathway only partially rescued these defects in HOIP-deficient thymocytes, suggesting additional roles for LUBAC in thymocyte differentiation.

By contrast, SHARPIN deficiency had a relatively mild impact on T-cell differentiation; SP maturation was normal but HELIOS upregulation in CD4SP was impaired, and numbers of Treg precursors and differentiated thymic FOXP3^+^CD25^+^ Treg cells markedly reduced. These distinct phenotypes are likely to reflect the relative roles of HOIP and SHARPIN in linear ubiquitination; loss of HOIP completely ablates linear ubiquitination following TNF stimulation, yet SHARPIN deficiency only partially impairs LUBAC activity (refs 17, 18, 19, our unpublished data). Thus, HOIL-1/HOIP complexes would sustain sufficient LUBAC function to support conventional thymocyte and NKT cell differentiation, yet optimal LUBAC activity (including SHARPIN) is necessary for the Treg cell sub-lineage, which is more heavily reliant on NF-κB signals^6^.

LUBAC is likely to coordinate signals from several stimuli essential for T-cell differentiation. Although a role for LUBAC in mediating B-cell receptor- and TCR-driven NF-κB signals via interactions with the CBM has been described^34^,^35^, the normal T-cell differentiation observed in CARD11-, BCL10- or MALT1-deficient mice (for example, see ref. 36) and our data showing that constitutive active IKK2 cannot restore peripheral T cells suggest that there are other LUBAC-dependent signals downstream of the TCR that are required for SP thymocyte maturation. Indeed, a role for SHARPIN has been implicated in JNK and ERK activation downstream of TCR signals^25^,^26^. Another possibility is that LUBAC is required to transduce signals from other cell surface receptors critical for SP maturation, such as members of the TNFR superfamily. This scenario may be particularly pertinent to the defects observed in HELIOS upregulation and thymic Treg differentiation. GITR, TNFR2 and OX40 play important, redundant roles in the intra-thymic differentiation of Treg cells that have received high affinity TCR signals^8^. Our data indicating that LUBAC has an important role in TNFR signalling in thymocytes support the notion that TNFR superfamily signals might also be important for the final stages of conventional T-cell differentiation in the thymus.

In this context, an important LUBAC function in several cell types is the inhibition of death receptor-mediated apoptosis or necroptosis. SHARPIN deficiency can predispose cells to caspase-8-dependent apoptosis or necroptosis, the latter via a pathway involving RIP3K and MLKL in cells receiving TNF signals^17^. Several lines of evidence suggest that induction of these cell death pathways does not account for the T-cell developmental defects we observed: (1) simultaneous genetic ablation of both of these cell death pathways did not rescue the impaired generation of thymic Treg cells in *Sharpin*^cpdm^ mice or the block in conventional thymocyte differentiation in *Hoip*^ΔCd4^ mice; (2) HOIL-1-, HOIP- and SHARPIN-deficient thymocytes were not predisposed to TNF-induced cell death; (3) pharmacologic inhibition of apoptosis or necroptosis did not alter thymocyte viability; and (4) TNF blockade *in vivo* did not rescue thymocyte differentiation in *Hoip*^ΔCd4^ mice or the loss of Treg cells in *Hoip*^ΔFoxp3^ mice. These data contrast recent findings that TNF deficiency could restore late thymocyte differentiation in IKK-deficient mice^51^ or TAK1-deficient mice^5^ and suggest roles for LUBAC beyond inhibiting TNF-induced cell death. In addition, the failure of combined BAX/BAK deletion to rescue SP thymocyte maturation in *Hoip*^ΔCd4^ mice provides evidence that LUBAC activity is not required to antagonize thymocyte deletion triggered by BH3-only proteins or to mediate cytokine-derived survival programmes (such as IL-7 or IL-2), stimuli that affect the mitochondrial pathway of apoptosis. Although we cannot exclude that alternative cell death pathways might be activated in LUBAC-deficient thymocytes, it is likely to be that LUBAC transduces other signals necessary for the transcriptional programmes guiding conventional T-cell and Treg cell differentiation in the thymus.

The partial rescue of thymocyte maturation observed in *Hoip*^ΔCd4^IKKca mice suggests that LUBAC-mediated NF-κB activation is important, but not sufficient to drive the final stages of thymocyte differentiation. Our transcriptional analysis comparing postpositive selection thymocytes from *Hoip*^ΔCd4^ and *Sharpin*^cpdm^ mice suggests a prominent role for pathways regulating cytokine responsiveness and metabolic fitness. Further studies will establish how LUBAC activity influences these pathways, but it is likely to be that this role also extends to peripheral Treg cell homeostasis. The deletion of HOIP following the thymic differentiation of FOXP3^+^ cells caused near-complete loss of peripheral Treg cells, establishing a cell intrinsic requirement for ongoing LUBAC activity in this lineage. The kinetics of the ensuing immunopathology was much swifter compared with that observed in mice lacking the pro-survival BCL-2 family member, MCL-1, specifically in Treg cells^52^, but was similar to that seen in *Foxp3*-deficient *scurfy* mutant mice^44^. This finding indicates an acute requirement for continued LUBAC-dependent signalling in Treg cells following their export to the periphery.

We conclude that LUBAC is essential for coordinating multiple signals required for the differentiation and homeostasis of conventional and non-conventional T-cell types required for adaptive immunity and tolerance.

## Methods

### Mice

The generation of *CD4*^*Cre*^, *Foxp3*^*Cre*^, *R26Stop*^*FL*^*ikk2ca*, *Bax*-floxed, *Bak*^−*/*−^ and *Rnf31*-floxed mice were previously described^18^,^37^,^45^,^53^,^54^,^55^. *Rbck1*^ΔCd4^, *Rnf31*^ΔCd4^, Rosa26-*ikk2ca*, *Rnf31*^ΔCd4^*Bax*^ΔCd4^*Bak*^−*/*−^, *Rnf31*^ΔCd4^*Casp8*^ΔCd4^*Mlkl*^−*/*−^, *Rnf31*^ΔFoxP3^, *Mlkl*^−/−^, *Mlkl*^−/−^*Casp8*^−/−^, *Sharpin*^cpdm^*Casp8*^−/−^*Mlkl*^−*/*−^, *Rip3*^−/−^*Casp8*^+/−^, *Sharpin*^cpdm^*Rip3*^−/−^*Casp8*^+/−^, *Tnf*^−*/*−^ and *Sharpin*^cpdm^*Tnf*^−*/*−^ mice were generated or backcrossed onto the C57BL/6 background from foundation strains. Rosa26-*ikk2ca* mice were obtained from Jackson Laboratories. *Sharpin*^cpdm^ mice on a C57BL/Ka background were obtained from Jackson Laboratories and were backcrossed onto the C57BL/6 background twice. All mice were housed at The Walter and Eliza Hall Institute of Medical Research (WEHI) under specific pathogen-free conditions. Experiments were performed in compliance with ethical regulations and were approved by the Animal Ethics Committee guidelines of the Melbourne Research Directorate.

### Generation of *Rbck1* floxed mice

The targeting construct was designed to introduce loxP sites on either side of a 1.5 kb genomic fragment containing *Rbck1* promoter and exons 1 and 2 (exon 2 contains the ATG start codon), as well as a FRT-flanked PGK-hygromycin resistance cassette for screening purposes (Supplementary Fig. 6). The targeting construct was electroporated into C57BL/6-derived Bruce-4 embryonic stem (ES) cells^56^. Homologous recombination events were identified by Southern blotting and blotting with a hygro-specific probe was used to confirm single-construct integration. A correctly targeted ES cell clone was injected into blastocysts, resulting in the gene-targeted mouse strain. The hygromycin-resistance cassette was deleted by crossing the resultant *Hoil*1-floxed heterozygous mice with C57BL/6-*flpe*-transgenic mice and the *flpe* transgene was subsequently eliminated by crossing offspring to C57BL/6 mice. All of the mice analysed were devoid of *hygro* and *flpe*. WT and floxed alleles were discriminated by a PCR with primers *Hoil*-Fwd 5′-ACCCTAGGCCTAGTCAGTGCAAA-3′ and Common-*Hoil*-Rev 5′-AGGCTGTGGTCCATTCTAGCCAT-3′ producing bands of 485 bp (WT) and 601 bp (floxed). The mutant allele after cre deletion was detected by PCR using Common-*Hoil*-Rev and *Hoil*-cre 5′-CCCTACAGCTAATTTTTGCAGATGTCAGG-3′ producing a 901 bp band. PCR conditions were: 95 °C 3 min, then 33 cycles 95 °C, 30 s, 55 °C, 20–30 s, 72 °C, 60 s.

### Bone marrow chimeras

To generate haematopoietic chimeras, C57/BL6.CD45.1/CD45.5.2 recipient mice were irradiated with two doses of 5.5 Gy given 3 h apart and then intravenously injected with 4 × 10^6^ T-cell-depleted bone marrow cells from CD45.2 experimental mice alone or mixed at a ratio of 1:1 with C57/BL6.CD45.1 WT bone marrow cells. Mice received 100 μg of anti-Thy1 mAb (clone T24) intraperitoneally 24 h after injection of bone marrow cells, to eliminate residual donor cells, then were allowed to reconstitute for 8–10 weeks before analysis.

### Flow cytometry

Single-cell suspensions of various lymphoid tissues were stained with fluorochrome or biotin conjugates, to detect the cell surface and intracellular proteins. Antibodies to cell surface proteins were purchased from BioLegend, except where indicated: CD4 (clone GK1.5), CD8 (clone 53-6.7), CD25 (clone PC61.5), TCRβ (clone H57-597), CD44 (clone IM7), CD62L (clone MEL-14), H2-K^b^ (clone AF6-88.5), CD69 (clone H1.2F3), CD24 (clone M1/69), CCR7 (clone 4B12), Helios (clone 22F6), TCRγδ (clone GL3), NK1.1 (clone PK136), B220 (WEHI, clone RA3-6B2), MHC II (WEHI, clone M15/114), Mac-1 (WEHI, clone M1/70), Gr-1 (WEHI, clone RB6-8C5), CD45.1 (WEHI, clone AL14A2), CD45.2 (clone 104), Ki-67 (BD Biosciences, clone BD56), CD152 (clone UC10-4B9), BCL2 (clone BCL/10C4) and FOXP3 (eBiosciences, clone FJK-16). Monomers of biotinylated mouse CD1d-PBS44 (a-GalCer analogue with a C24:1 acyl chain were tetramerized with streptavidin conjugated to phycoerythrin (BD Pharmingen). Staining for CCR7 was done for 60 min at 37 °C in pre-warmed FACS buffer (PBS containing 1% vol/vol heat-inactivated bovine serum and 5 mM EDTA). All other surface stains were incubated for 30 min at 4 °C. Intracellular staining for FOXP3, Ki67, BCL2 or Helios was performed after fixation and permeabilization using the reagents from the eBiosciences FOXP3 staining kit. Sample data were acquired on an LSRII or Fortessa flow cytometer (BD Biosciences) and analysed using FlowJo software (TreeStar).

### Western blotting

Unfractionated thymocytes were stimulated for various periods of time with plate-bound monoclonal 10 μg ml^−1^ anti-CD3 (WEHI Monoclonal Antibody Facility, clone 145-2C11)+10 μg ml^−1^ anti-CD28 (WEHI, clone 37N51) or 100 ng ml^−1^ Fc-TNF (WEHI). Cells were washed twice in PBS before lysate preparation. Cell lysates were prepared in DISC buffer (1% NP-40, 10% glycerol, 150 mM NaCl, 20 mM Tris pH 7.5, 2 mM EDTA, Roche complete protease inhibitor cocktail, 2 mM sodium orthovanadate, 10 mM sodium fluoride, β-glycerophosphate and N_2_O_2_PO_7_). Cell lysates were loaded in NuPAGE Bis-Tris gels (Life Technologies, Mulgrave, VIC, Australia) and transferred onto Immobilon-P poly(vinylidene difluoride) membranes (Millipore, Billerica, MA, USA) or Hybond-C Extra (GE Healthcare). Membranes were blocked and antibodies were diluted in 5% skimmed milk powder or BSA in 0.1% Tween-20/NaCl/P_i_ or NaCl/Tris. Antibodies against phospho-p65 (Cell Signalling Technologies, clone 93H1, 1:1,000), phospho-p38 (Cell Signalling Technologies, clone D3F9, 1:1,000), IκBα (Cell Signalling Technologies, cat number #9242, 1:1,000), β-actin (Sigma Aldrich, clone AC-15, 1:5,000) were used for western blotting. Signals were detected by chemiluminescence (Millipore) after incubation with secondary antibodies conjugated to horseradish peroxidase. Images have been cropped for presentation. Full-sized images are presented in Supplementary Fig. 7.

### *In vitro* cell death assay

Cells cultured in 96-well tissue culture plates were harvested 24 h after stimulation with Fc-TNF (WEHI)/SMAC-mimetic Compound A (GT12911, TetraLogic Pharmaceuticals) with or without QVD-OPh (MP Biomedicals)/necrostatin-1 (TetraLogic Pharmaceuticals) and cell death was measured by propidium iodide (PI) staining. Flow cytometry was performed on a LSRII or Fortessa flow cytometer (BD Biosciences) and data were analysed using FlowJo software (TreeStar).

### Measurement of serum IgE

Serum IgE (1:10 dilution) was measured by ELISA using 2 μg ml^−1^ rat anti-mouse Ig antibodies (Southern Biotech, clone 23G3) as a capture reagent and developed with 1:500 mouse Ig isotype-specific goat anti-mouse IgE (Fc specific) conjugated to horseradish peroxidase (Nordic MUBio)^57^. Capture reagent was applied overnight at 4 °C, sera was applied for 4 h at room temperature and ELISA plates were developed in the dark for 45 min at room temperature. IgE isotype monoclonal anti-dinitrophenyl antibody produced in mouse, IgE isotype (Sigma, MO, clone SPE-7) were used as standards.

### RNA sequencing

Thymocytes were prepared from three mice of each genotype (WT, *Hoil*^ΔCd4^ and *Sharpin*^cpdm^), and CD69^+^ MHC I^low^ and CD69^+^ MHC I^high^ cells were FACS purified on a MoFlo cell sorter (Beckman Coulter), with a dump channel to gate out PI^+^, CD25^+^, CD44^+^, NK1.1^+^, B220^+^, MHC II^+^, Gr1^+^, Mac-1^+^ and δTCR^+^ cells. Sorted cells were preserved in RNAlater (ThermoFisher Scientific) and frozen at −80 **°**C, then RNA was isolated with RNeasyPlus Mini kit (Qiagen). Messenger RNA reverse transcription and complementary DNA libraries were prepared using the TruSeq RNA Sample preparation kit (Illumina) following the manufacturer's instructions. Indexed sample libraries were subjected to 75 base single-end sequencing using the 75 cycle high-output kit v2 chemistry for the NextSeq 500 sequencing instrument (Illumina).

### Bioinformatics analysis

Sequencing reads were mapped to the mouse genome (mm10) using the subread aligner^58^ implemented in the Rsubread software package. Gene-level read counts were obtained using featureCounts^59^ and its inbuild mm10 annotation, which includes Entrez gene ID, chromosome and gene length information, corresponding to the NCBI RefSeq annotations. Gene annotation was obtained from the NCBI Mus musculus gene info file (downloaded 25 September 2015). Statistical analysis used the edgeR^60^ and limma^61^ software packages. Genes were filtered as not expressed if they failed to show at least 0.5 count per million reads in at least 3 samples. As there are female and male mice in this experiment, genes from chromosome Y and the gene Xist were filtered out so as to correct for gender effect. Predicted genes and the genes without official gene symbols were also filtered out. TMM scale normalization^62^ was applied and read counts were transformed to log2 counts per million with a prior count of 1 using the edgeR cpm function. Linear models were used to test for expression differences between different genotypes. Empirical array quality weights were estimated to allow for differences in quality between the RNA samples^63^. Each mouse was treated as a random block, allowing for correlation between CD69^+^ MHC I^low^ and CD69^+^ MHC I^high^ cells from the same mouse^64^. Differential expression between genotypes were assessed using empirical Bayes moderated *t*-statistics, allowing for an abundance trend in the s.e. and for robust estimation of Bayesian hyperparameters^65^. The Benjamini and Hochberg method^66^ was used to adjust the *P*-values so as to control the false discovery rate.

A list of NF-κB target genes was obtained from http://www.bu.edu/nf-kb/gene-resources/target-genes. A number of steps were required to convert the various gene identifiers to mouse Entrez Gene IDs. Where possible, gene aliases were converted to current official human gene symbols using the Bioconductor annotation package, org.Hs.eg.db. Otherwise, human RefSeq accession numbers were converted to human symbols using org.Hs.eg.db and mouse RefSeq accession numbers were converted to mouse Entrez Gene IDs using org.Mm.eg.db. Finally, human symbols were mapped to mouse Entrez Gene IDs using the Jackson Laboratory mouse–human orthologue table downloaded December 2012 (ref. 67) and the NCBI mouse–human homologue table downloaded August 2013. NF-κB target genes that were differentially expressed at 5% false discovery rate between CD69^+^ MHC I^high^ versus CD69^+^ MHC I^low^ cells in WT mice were then used for gene-set testing between CD69^+^ MHC I^high^ versus CD69^+^ MHC I^low^ in Sharpin and between CD69^+^ MHC I^high^ versus CD69^+^ MHC I^low^ in HOIL-1. Gene-set testing was conducted using limma's roast function^68^, with 9,999 residual rotations and the same linear model settings as for the differential expression analysis. Barcode enrichment plots were produced using limma's barcode plot function.

### Statistical analysis

Statistical comparisons were made using one-way analysis of variance with a Tukey's *post-hoc* test for multiple comparisons with Prism v.6.0 (GraphPad). *P*-values <0.05 were considered to indicate a statistically significant difference.

### Data availability

Sequence data that support the findings of this study have been deposited in GEO with the primary accession code GSE74552. All additional data supporting the findings of this study are available within this article and its Supplementary Information files or from the corresponding author on a reasonable request.

## Additional information

**How to cite this article:** Teh, C. E. *et al*. Linear ubiquitin chain assembly complex coordinates late thymic T-cell differentiation and regulatory T-cell homeostasis. *Nat. Commun.* **7,** 13353 doi: 10.1038/ncomms13353 (2016).

**Publisher's note:** Springer Nature remains neutral with regard to jurisdictional claims in published maps and institutional affiliations.

## Supplementary Material

Supplementary Information Supplementary Figures 1-7
