# Supplementary material for: Linear ubiquitin chain assembly complex coordinates late thymic T-cell differentiation and regulatory T-cell homeostasis
Source: Nat Commun. 2016 Nov 18;7:13353. doi: 10.1038/ncomms13353 (PMC5120208; doi:10.1038/ncomms13353)
Supplement: Supplementary Information — Supplementary Figures 1-7 [file ncomms13353-s1.pdf]

SUPPLEMENTARY INFORMATION

Supplementary Figures

Supplementary Figure 1

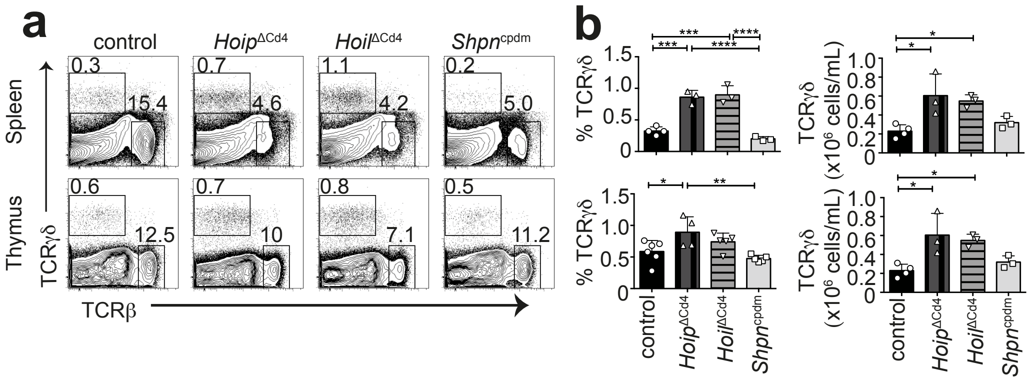

Supplementary Figure 1. (**A**) Surface expression of TCRδ and TCRβ on splenocytes (top panel) and thymocytes (bottom panel) of 7-15 week-old control, *Hoip*$^{\Delta Cd4}$, *Hoil*$^{\Delta Cd4}$ and *Sharpin*^{cpdm} mice. (**B**) Percentages (left panels) and total numbers (right panels) of TCRγδ cells in spleen (top) and thymus (bottom) of control, *Hoip*$^{\Delta Cd4}$, *Hoil*$^{\Delta Cd4}$ and *Sharpin*^{cpdm} mice. Each symbol represents an individual mouse; small horizontal lines indicate mean±s.d.; *, **, *** and **** denotes $p<0.05$, $p<0.01$, $p<0.005$ and $p<0.001$, respectively. One-way ANOVA with a Tukey's post hoc test for multiple comparisons was used for statistical analysis. *Shpn*^{cpdm} refers to *Sharpin*^{cpdm} mice. Data are representative of 2 independent experiments with 4-6 mice per group.

Supplementary Figure 2.

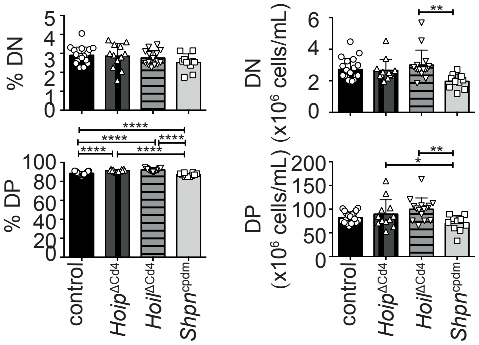

Supplementary Figure 2. Quantification of total cell numbers and percentages of DN ($CD4^-CD8^-$) and DP ($CD4^+CD8_+$) thymocytes from control, *Hoip*$^{\Delta Cd4}$, *Hoil*$^{\Delta Cd4}$ and *Sharpin*^{cpdm} mice. Each symbol represents an individual mouse; small horizontal lines indicate mean±s.d.; *, **, *** and **** denotes $p<0.05$, $p<0.01$, $p<0.001$ and $p<0.005$, respectively. One-way ANOVA with a Tukey's post hoc test for multiple comparisons was used for statistical analysis. *Shpn*^{cpdm} refers to *Sharpin*^{cpdm} mice. Data are pooled from 6 independent experiments with 2-6 mice per group.

Supplementary Figure 3.

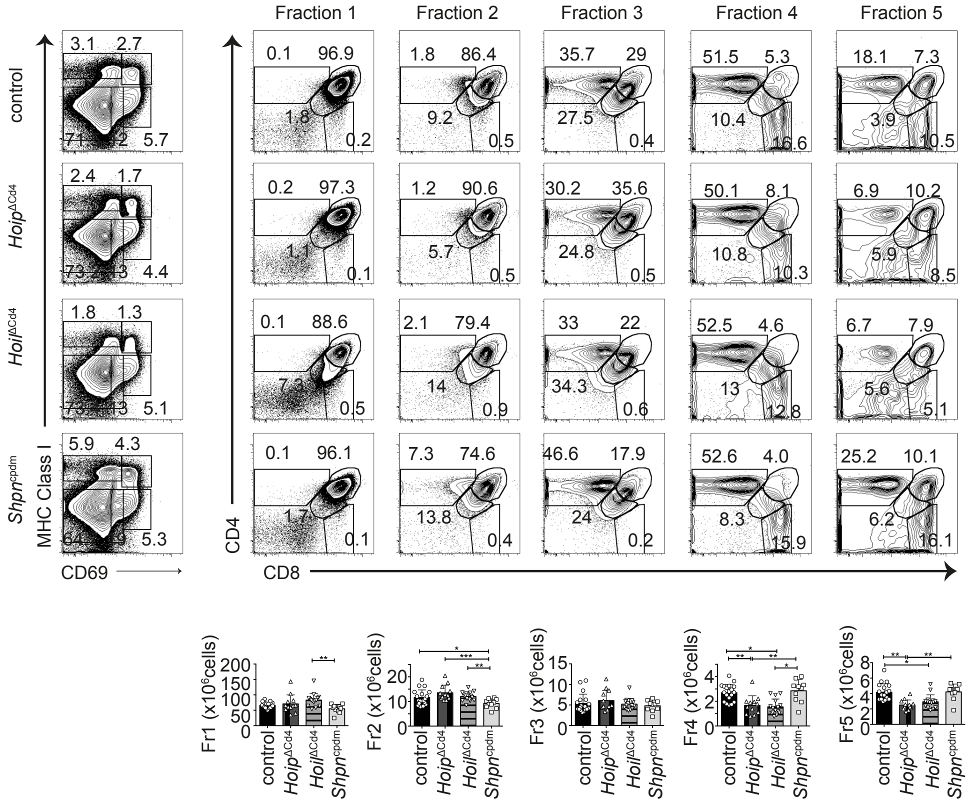

Supplementary Figure 3. Expression of MHC Class I (H2-K^b) and CD69 on total control, *Hoip*$^{\Delta Cd4}$, *Hoil*$^{\Delta Cd4}$ and *Sharpin*^{cpdm} thymocytes analyzed by flow cytometry, delineating five fractions. The corresponding surface expression of CD4 and CD8 on fractions 1-5 is indicated. The fractions are enumerated in the bar graphs below, with each symbol representing an individual mouse; small horizontal lines indicate mean±s.d.; *, **, *** and **** denotes $p<0.05$, $p<0.01$, $p<0.005$ and $p<0.001$, respectively. One-way ANOVA with a Tukey's post hoc test for multiple comparisons was used for statistical analysis. *Shpn*^{cpdm} refers to *Sharpin*^{cpdm} mice. Data are pooled from 6 independent experiments with 2-6 mice per group.

Supplementary Figure 4

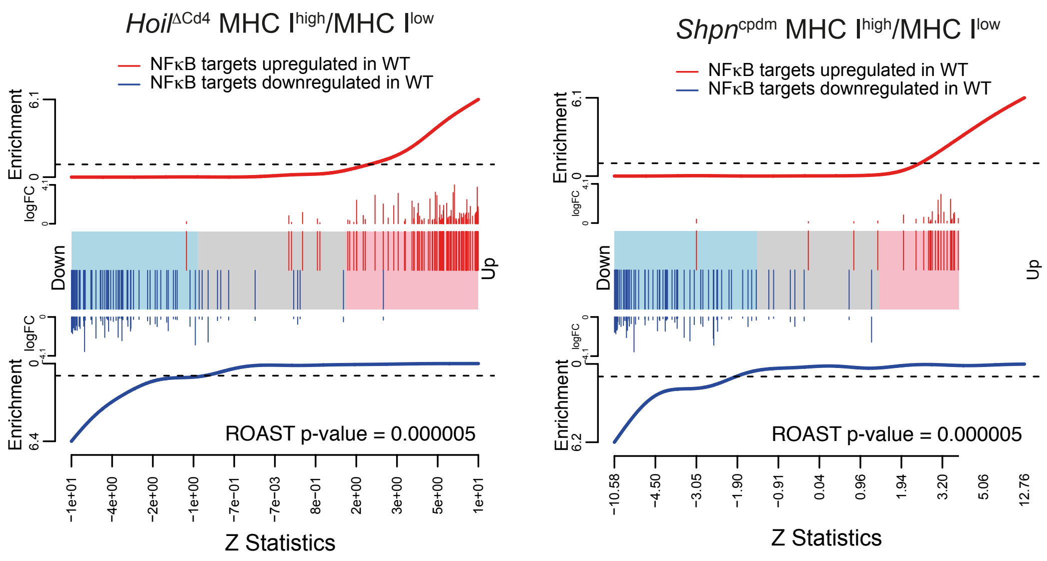

Supplementary Figure 4. Barcode plots showing that the transcriptional profile of NF-κB target genes for MHC I^{high} vs MHC I^{low} $CD69^{+}$ thymocytes in WT mice is preserved in *Hoil*$^{\Delta Cd4}$ (right) and *Sharpin*^{cpdm} (left) mice. In each plot, all genes in the genome are ordered from most up-regulated in MHC I^{high} (right) to most down-regulated in MHC I^{high} (left), in the mutant mice. Vertical bars show the rank positions of NF-κB genes that are up-regulated (red) or down-regulated (blue) for the same comparison in WT. The log2-fold-change of each gene in WT is also shown. The worms show relative enrichment.

Supplementary Figure 5

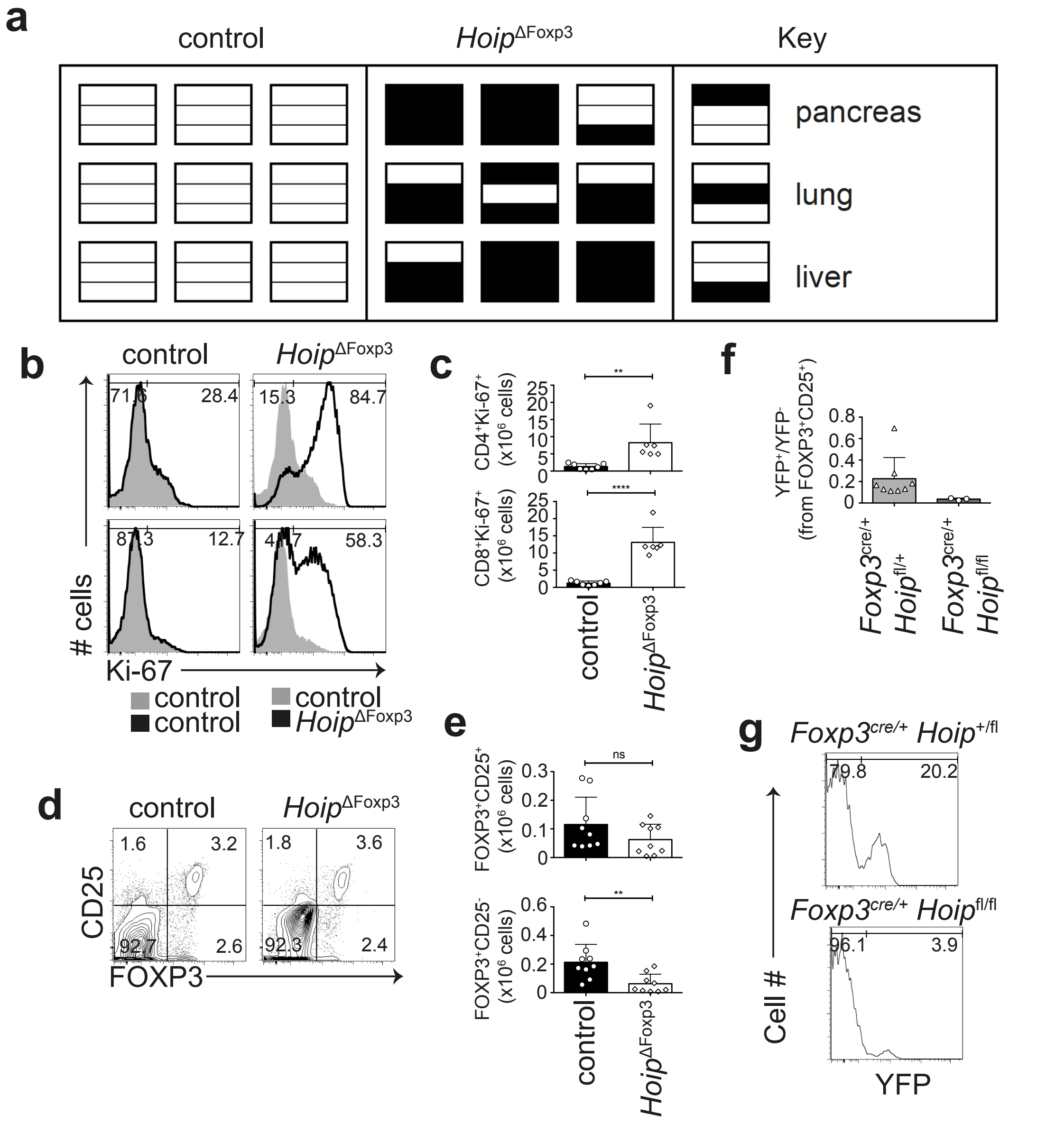

Supplementary Figure 5. (**a**) Summary of immune infiltration detected in the pancreas, lung and liver for *Hoip*$^{\Delta Foxp3}$ mice and littermate controls. Each sector represents the detection of lymphocytic infiltration in a single mouse and shaded sections denote the presence of infiltration. (**b**) Expression of Ki-67 on $CD4^+FOXP3^-$ (top panel) and $CD8^+$ (bottom panel) cells and (**c**) absolute

numbers of Ki-67^{high} cells in the spleen from wild-type (grey shaded histogram) and *Hoip*$^{\Delta Foxp3}$ (black thick histogram) mice. (**d**) CD25 and FOXP3 expression of thymic CD4^{+} cells from *Hoip*$^{\Delta Foxp3}$ mice. (**e**) Number of thymic FOXP3^{+}CD25^{+} Treg cells. (**f**) Ratio of YFP^{+}/YFP^{-} cells on CD4^{+}CD25^{+} cells in the blood of *Foxp3*$^{cre/+}$*Hoip*$^{+/fl}$ and *Foxp3*$^{cre/+}$*Hoip*$^{fl/fl}$ mice. (**g**) Representative histograms of YFP expression in CD4^{+}CD25^{+} cells in the spleen of *Foxp3*$^{cre/+}$*Hoip*$^{+/fl}$ and *Foxp3*$^{cre/+}$*Hoip*$^{fl/fl}$ mice. *Shpn*^{cpdm} refers to *Sharpin*^{cpdm} mice. Data are representative of 3 independent experiments with 1-3 mice per group (**a**-**e**) or 1 experiment with 3-8 mice per group (**f**-**g**). One-way ANOVA with a Tukey's post hoc test for multiple comparisons was used for statistical analysis.

Supplementary Figure 6

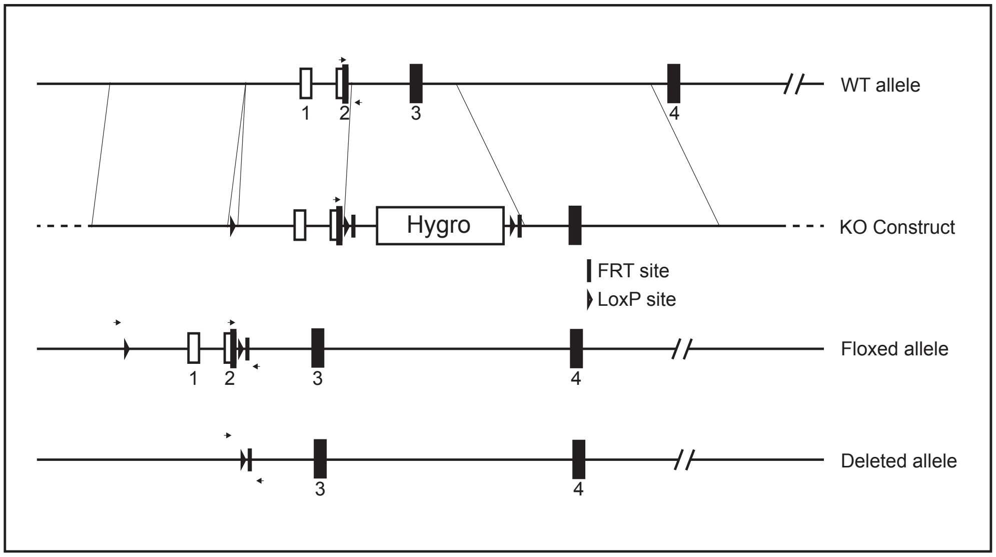

Supplementary Figure 6. Targeting strategy and generation of *Hoil-1* (*Rbck1)* floxed mice. Schematic diagram depicting the *Rbck1* locus targeting strategy. Open boxes represent non-coding parts of exons 1 and 2, black boxes represent coding part of exons 2, 3 and 4. Targeting construct contains a hygromycin resistance selection cassette (FRT/pGK-Hygro/LoxP/FRT), whereas triangles and small rectangles indicate the loxP sites and frt sites, respectively. Arrowheads indicate the positions of the primers used for genotyping.

Supplementary Figure 7

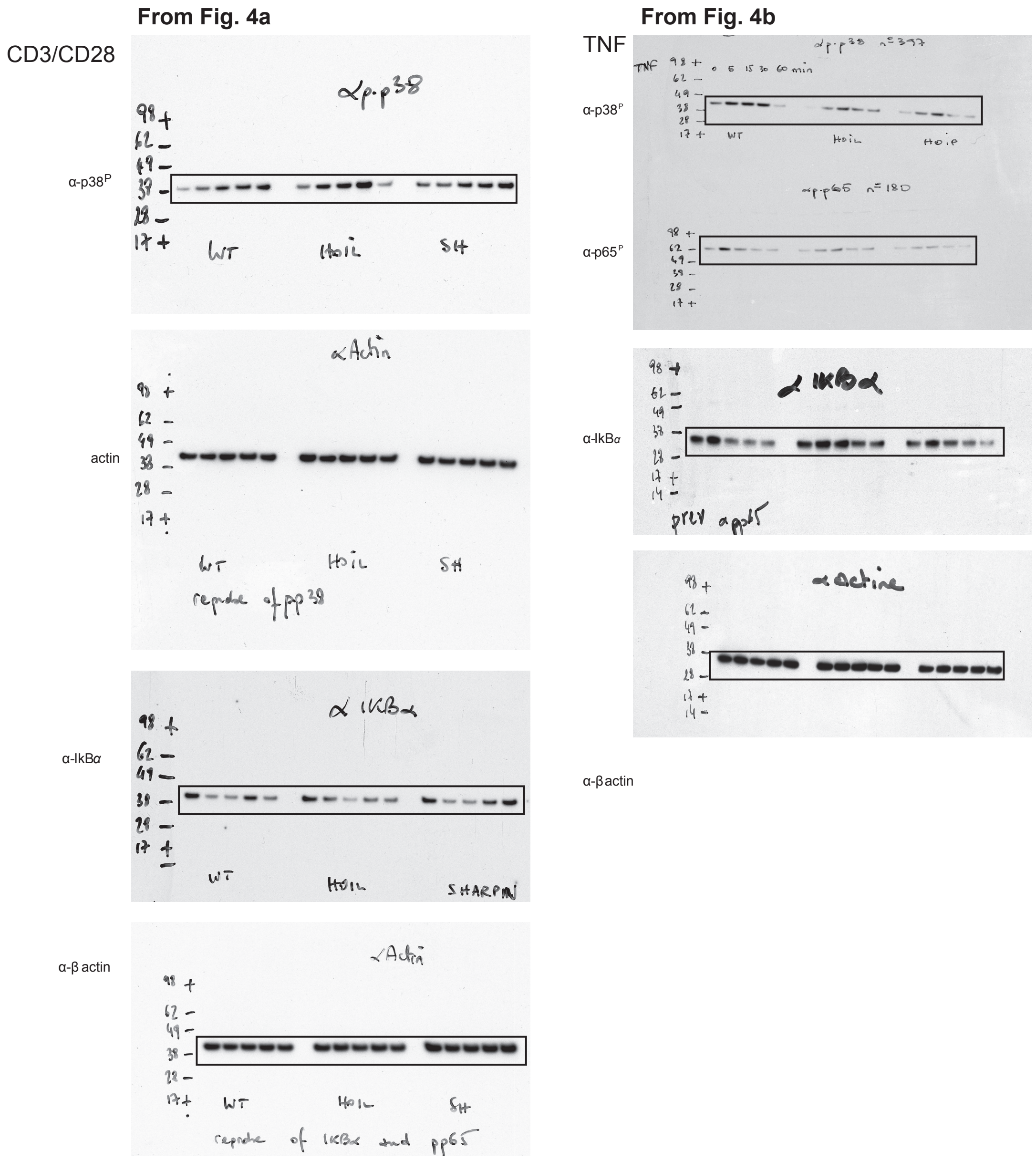

Supplementary Figure 7. Original Western blots images used in Figure 4a and b. Blocks indicate specific bands in the main figure.
